# Whole-genome sequencing for prediction of *Mycobacterium tuberculosis* drug susceptibility and resistance: a retrospective cohort study

**DOI:** 10.1016/S1473-3099(15)00062-6

**Published:** 2015-10

**Authors:** Timothy M Walker, Thomas A Kohl, Shaheed V Omar, Jessica Hedge, Carlos Del Ojo Elias, Phelim Bradley, Zamin Iqbal, Silke Feuerriegel, Katherine E Niehaus, Daniel J Wilson, David A Clifton, Georgia Kapatai, Camilla L C Ip, Rory Bowden, Francis A Drobniewski, Caroline Allix-Béguec, Cyril Gaudin, Julian Parkhill, Roland Diel, Philip Supply, Derrick W Crook, E Grace Smith, A Sarah Walker, Nazir Ismail, Stefan Niemann, Tim E A Peto, Modernizing Medical Microbiology (MMM) Informatics Group

**Affiliations:** a Nuffield Department of Medicine, University of Oxford, John Radcliffe Hospital, Oxford, UK; b Molecular Mycobacteriology, Forschungszentrum Borstel, Leibniz-Zentrum für Medizin und Biowissenschaften, Borstel, Germany; c Centre for Tuberculosis, National Institute for Communicable Diseases, Johannesburg, South Africa; d Wellcome Trust Centre for Human Genetics, University of Oxford, Oxford, UK; e German Center for Infection Research, Borstel Site, Borstel, Germany; f Institute of Biomedical Engineering, Department of Engineering Science, University of Oxford, Oxford, UK; g Microbiology Services, Public Health England, London, UK; h Public Health England National Mycobacterial Reference Laboratory, Queen Mary's School of Medicine and Dentistry, London, UK; i Department of Infectious Diseases, Imperial College, London, UK; j Genoscreen, Lille, France; k Wellcome Trust Sanger Institute, Hinxton, UK; l Institute for Epidemiology, University Medical Hospital Schleswig-Holstein, Airway Research Center North, Kiel, Germany; m Centre National de la Recherche Scientifique, Lille, France; n INSERM, Université de Lille, and Campus de l'Institut Pasteur de Lille, Center for Infection and Immunity of Lille, Lille, France; o National Institute of Health Research Oxford Biomedical Research Centre, John Radcliffe Hospital, Oxford, UK; p Public Health England West Midlands Public Health Laboratory, Heartlands Hospital, Birmingham, UK; q Department of Medical Microbiology, University of Pretoria, Pretoria, South Africa

## Abstract

**Background:**

Diagnosing drug-resistance remains an obstacle to the elimination of tuberculosis. Phenotypic drug-susceptibility testing is slow and expensive, and commercial genotypic assays screen only common resistance-determining mutations. We used whole-genome sequencing to characterise common and rare mutations predicting drug resistance, or consistency with susceptibility, for all first-line and second-line drugs for tuberculosis.

**Methods:**

Between Sept 1, 2010, and Dec 1, 2013, we sequenced a training set of 2099 *Mycobacterium tuberculosis* genomes. For 23 candidate genes identified from the drug-resistance scientific literature, we algorithmically characterised genetic mutations as not conferring resistance (benign), resistance determinants, or uncharacterised. We then assessed the ability of these characterisations to predict phenotypic drug-susceptibility testing for an independent validation set of 1552 genomes. We sought mutations under similar selection pressure to those characterised as resistance determinants outside candidate genes to account for residual phenotypic resistance.

**Findings:**

We characterised 120 training-set mutations as resistance determining, and 772 as benign. With these mutations, we could predict 89·2% of the validation-set phenotypes with a mean 92·3% sensitivity (95% CI 90·7–93·7) and 98·4% specificity (98·1–98·7). 10·8% of validation-set phenotypes could not be predicted because uncharacterised mutations were present. With an in-silico comparison, characterised resistance determinants had higher sensitivity than the mutations from three line-probe assays (85·1% *vs* 81·6%). No additional resistance determinants were identified among mutations under selection pressure in non-candidate genes.

**Interpretation:**

A broad catalogue of genetic mutations enable data from whole-genome sequencing to be used clinically to predict drug resistance, drug susceptibility, or to identify drug phenotypes that cannot yet be genetically predicted. This approach could be integrated into routine diagnostic workflows, phasing out phenotypic drug-susceptibility testing while reporting drug resistance early.

**Funding:**

Wellcome Trust, National Institute of Health Research, Medical Research Council, and the European Union.

## Introduction

WHO's target is to end the tuberculosis epidemic by 2035. Multidrug-resistant tuberculosis poses the greatest obstacle to success, with an estimated 480 000 cases worldwide in 2013 alone.^1^ Phenotypic drug-susceptibility testing for *Mycobacterium tuberculosis* can take many weeks, and access to the necessary laboratory facilities in countries with the greatest disease burden is often scarce.^1^ Although genotypic assays are faster and have diagnostic usefulness in both high-income and low-income countries,^2^, ^3^, ^4^ these assays screen a small number of genetic loci commonly associated with drug resistance, but are not designed to identify or exclude resistance by other mechanisms.^5^, ^6^ Culture-based drug-susceptibility testing thus remains the gold-standard assay for testing resistance.

Whole-genome sequencing enables the screening of known resistance-associated loci while also providing opportunities to characterise other loci as predictive of resistance or not.^2^, ^7^, ^8^ To assess whether data from whole-genome sequencing can be used clinically to predict both drug resistance and drug susceptibility, we characterised the genetic variation in a large training set of samples and validated the findings by predicting phenotypes in an independent dataset.

## Methods

### Sample selection and processing

We included 3651 *M tuberculosis* complex genome sequences from the UK, Sierra Leone, South Africa, Germany, and Uzbekistan, representing all seven global clades (appendix 1).^9^ We did phenotypic drug-susceptibility testing at reference laboratories in each of the countries (appendix 1) using the WHO-endorsed proportion method in an automated Mycobacterial Growth Indicator Tube 960 system (Becton Dickinson), on solid Lowenstein-Jensen media, or the resistance ratio method. UK samples were tested for routine patient-care purposes, and non-UK samples for research. Drug-susceptibility testing for one or more of isoniazid, rifampicin, ethambutol, pyrazinamide, streptomycin, ciprofloxacin, moxifloxacin, ofloxacin, amikacin, capreomycin, and kanamycin was available for each isolate. We prepared DNA for sequencing using the Nuclisens EasyMag (Biomerieux, France) following the manufacturer's protocol, the Fuji Quickgene kit (Kurabo Biomedical, Osaka, Japan), or the cetyltrimethylammonium bromide method of DNA purification (as previously described).^10^, ^11^ We used Illumina (San Diego, CA, USA) sequencing platforms at the Wellcome Trust Centre for Human Genetics (Oxford, UK), the Wellcome Trust Sanger Institute (Hinxton, UK), the Forschungszentrum Borstel (Borstel, Germany); Genoscreen (Lille, France), and the National Institute for Communicable Diseases (Johannesburg, South Africa).

**Research in context**

**Evidence before this study**

We searched the PubMed database for studies published before April, 2015, using the terms “whole genome sequencing”, “tuberculosis”, “drug resistance”, “drug susceptibility”, “prediction”, and “discovery”. Much of the scientific literature on drug resistance in tuberculosis up to 2010 has concentrated on identifying resistance-conferring mutations, and has been summarised in the Tuberculosis Drug Resistance Mutation Database. This database includes the small number of common drug-resistance mutations upon which the design of commercial molecular assays such as the Hain MTBDRplus line-probe (Nehren, Germany) and the Cepheid MTB/RIF GeneXpert (Sunnyvale, CA, USA) are based. As these assays only screen for phenotypic resistance, and not phenotypic susceptibility, expensive and slow phenotypic drug-susceptibility testing remains necessary to define which drugs will effectively treat patients. Some studies have used DNA sequencing techniques to predict phenotypic resistance from a wider set of known genotypic-resistance mechanisms, and to discover new drug-resistance mechanisms. One recent study made an important contribution by seeking to characterise each mutation in the *pncA* gene, relevant to the key first-line drug pyrazinamide, as either conferring resistance or not conferring resistance, thereby raising the prospect of predicting both drug resistance and drug susceptibility from genetic data, and reducing the need for phenotyping for pyrazinamide.

**Added value of this study**

Our study expands on these findings by examining mutations for all first-line and second-line antituberculosis drugs. By using a large number of whole-genome sequences we were able to control for population structure and characterise mutations within relevant genes identified in the scientific literature as either conferring resistance or consistent with drug susceptibility. We are also able to search the rest of the genome for additional genes of relevance to drug resistance. Through characterisation of all mutations, this approach can establish which drugs will be effective against clinical isolates and, because it is iteratively updatable, can result in fewer isolates needing phenotypic drug-susceptibility testing.

**Implications of all the available evidence**

The growing body of knowledge on mutations conferring drug resistance or consistent with susceptibility will provide the basis from which a near-definitive genotypic assay can be designed that will eventually bypass the need for phenotypic drug-susceptibility testing. Early results from this study have provided information for a pilot of drug-susceptibility testing based on whole-genome sequencing in the UK, and, as portable whole-genome sequencing platforms become available, could transform drug-susceptibility testing in low-income settings where many of the world's patients with tuberculosis live, and where many still rely on empirical treatment regimens.

Paired-end reads were mapped with Stampy^12^ (version 1.0.17) to the H37Rv (GenBank NC000962.2) reference genome, which was phenotypically susceptible to all drugs of interest. Repetitive genome sections were defined by self-self BLAST and masked. We excluded isolates with less than 88% mapped coverage of the reference genome (appendix 1). Base calls were made with SAMtools mpileup^13^ (version 0.1.18), requiring a minimum-read depth of 5 ×, including at least one read on each strand. Where an alternative base represented more than 10% of read depth, mixed base calls were made. These base calls were only included in the downstream analysis if, in at least one other isolate, they constituted more than 90% of read depth (ie, a non-mixed base call). We identified insertions and deletions with Cortex.^14^ One inconsistent base call was identified across 202 technical replicates (error <1 × 10^−9^ per base). We used RAxML (version 8.0.5) to reconstruct the phylogeny under a general time reversible model with rate variation modelled by fixed-rate categories.^15^ We estimated the frequency of each single nucleotide polymorphism arising in the phylogeny (ie, homoplasy) using maximum-likelihood ancestral site reconstruction.^16^ No ethics approval was required for this study.

### Characterisation of mutations

**Figure 1:**
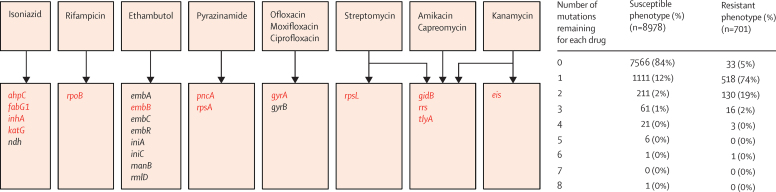
Candidate genes and mutations The number of potentially predictive mutations in genes relevant to each drug after lineage-defining and synonymous mutations have been set aside and are shown by susceptible and resistant phenotypes for 2099 training-set isolates. Genes from which one or more of the 120 resistance-determining mutations were algorithmically characterised are coloured red.

Identification of resistance-causing single-nucleotide polymorphisms in clonal bacteria using genome-wide association studies is challenging.^17^ We therefore first focused on 23 candidate genes and their promoter regions (figure 1), each with at least one previously described drug-resistance mutation (appendix 1). We devised an algorithm to characterise all mutations for these genes compared with the pan-susceptible reference genome at the level of single-nucleotide polymorphisms in promoter regions, aminoacids in genes, or insertions and deletions. We characterised mutations separately for each relevant drug without taking previous findings about specific mutations from the scientific literature into account, and then used these results to predict drug-susceptibility test results in other independent samples.

Samples were obtained in batches over time. We first used a training set of 2099 samples from the UK, Sierra Leone, and South Africa to characterise mutations as resistance determining or not. We then obtained a validation set of 1552 samples from Germany, Uzbekistan, and South Africa, against which characterisations were assessed. To check for bias resulting from the composition of sets, we repeated analyses after switching training and validation sets, and then repeated a further 100 times, randomly allocating samples to equally sized training and validation sets (appendix 1).

To algorithmically characterise mutations in the training set, we first assumed that synonymous and lineage-defining mutations do not cause resistance, unless the lineage-defining mutations were associated with lineage-specific resistance (eg, pyrazinamide in *Mycobacterium bovis;*^18^ appendix 1). After these mutations were labelled as benign and set aside, we assessed the remaining mutations within each group of genes relevant to each drug in turn, and generated hypotheses regarding associations with drug resistance (to be tested in the validation set). A mutation was characterised as resistance determining if it occurred as the only mutation across all relevant candidate genes in at least one phenotypically resistant isolate in the training set. Mixed-base calls were regarded as mutations rather than wild types. Since mutations that do not cause resistance can clearly co-occur with those that do, mutations were characterised as benign if they take place only in phenotypically susceptible isolates, or where all isolates were phenotypically susceptible when a mutation occurred alone. These benign mutations were then also set aside and the analysis repeated to potentially reveal further resistance-determining mutations (appendix 1). Where resistance could not be accounted for by a characterised resistance determinant, evidence of synergy between mutations, or of co-occurring compensatory mutations, was sought by manual inspection of sequences.

Validation-set isolates containing mutations characterised in the training set as resistance determining were predicted resistant, and those containing no mutations, or only mutations characterised as benign, were predicted susceptible. Isolates containing uncharacterised mutations were hence not predicted unless co-occurring with resistance determinants. We then made a comparison with predictions based only on mutations probed by the Genotype MTBDRplus, MTBDRsl (HAIN Life-sciences, Germany), and AID (AID Diagnostika, Germany) line-probe assays. Finally, all 3651 isolates were combined and the algorithm reapplied.

Because some resistant phenotypes might not be attributable to mutations in the 23 genes, the remaining genome was explored for potential explanatory mutations. Because resistance-causing mutations are likely to be under positive selection pressure, these mutations are also the most likely to arise repeatedly, independently in the phylogeny.^8^ Focusing our search for additional resistance determinants on these homoplasic mutations, we quantified the frequency of homoplasic events for each mutation in the genome and compared the frequency recorded across the 23 genes and among characterised resistance determinants to that among mutations in genes or open reading frames and functional RNA molecules elsewhere in the genome.^19^ Analyses were done with Stata 13.1 (StataCorp, Texas).

### Role of the funding source

The funder of the study had no role in study design, data collection, data analysis, data interpretation, or writing of the report. The corresponding author had full access to all the data in the study and had final responsibility for the decision to submit for publication.

## Results

2099 *M tuberculosis* isolates were sequenced as a training set, within which 1414 independent strains could be identified by clustering isolates within five single-nucleotide polymorphisms of another.^11^ 382 (18·2%) were phenotypically resistant to at least one drug, 91 (4·3%) were multidrug resistant, and four (0·2%) were extensively drug resistant, making a total of 701 (7·2%) resistant phenotypes. 8978 susceptible phenotypes were identified.

The individual steps in the mutation identification algorithm are detailed in appendix 1. After all lineage-defining and synonymous mutations were set aside, apart from *pncA* H57D and *rpsA* A440T because these were present in all *M bovis* isolates (intrinsically pyrazinamide resistant), 991 mutations (counting mutations more than once if relevant to more than one drug) were left for consideration. One mutation remained in 518 (74%) of resistant and 1111 (12%) of susceptible phenotypes, whereas no mutations remained for 7566 (84%) of susceptible and 33 (5%) of resistant phenotypes (figure 1). 112 mutations were thereby classified as resistance determining, and 772 were classified as benign. After setting these benign mutations aside, six additional mutations were classified as resistance determining, but no further mutations could be characterised by repeating the algorithm again. 101 mutations thus remained unclassified, of which 60 co-occurred only with resistance determinants, compatible with a possible compensatory role (appendix 1). The 120 resistance-determining mutations (including *pncA* H57D and *rpsA* A440T) were spread across just 14 candidate genes (figure 1), with 79 (66%) of 120 having previously been described as resistance determining in the scientific literature (appendix 1).

At least one resistance determinant was present in 658 (93·9%) of 701 resistant training-set phenotypes. 33 (4·7%) of 701 resistant phenotypes remained unaccounted for with no relevant mutations in relevant genes, and 10 (1·4%) of 701 could not be algorithmically unravelled because they contained more than one relevant mutation. Six of these contained mutations associated with resistance in the scientific literature (appendix 1).

We also noted resistance-determining mutations in 121 susceptible phenotypes. Such phenotypic variability was most evident for isolates containing *embB* M306I and *rpoB* I491F. 34 (68%) of 50 containing *embB* M306I were phenotypically susceptible to ethambutol and 19 (83%) of 23 containing *rpoB* I491F were phenotypically susceptible to rifampicin (appendix 1). Although mutations elsewhere in the genome might account for such variability through epistasis, a subset of eight ethambutol-resistant and three rifampicin-resistant isolates each had a genetically indistinguishable (ie, no single-nucleotide polymorphisms) but phenotypically susceptible paired isolate. Such phenotypic changes without genotypic changes suggest poor phenotypic reproducibility for these mutations at least.^20^

**Figure 2:**
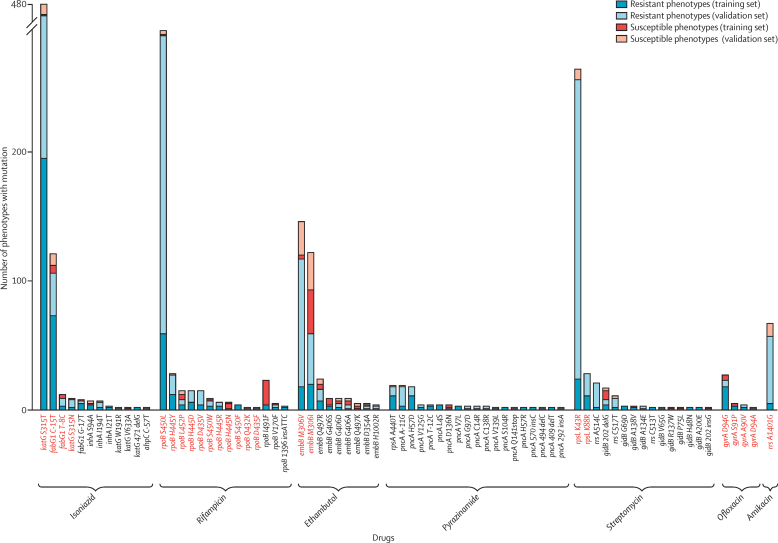
Resistance determinants in training and validation sets Mutations probed by a line-probe assay are coloured red. Mutations that were only noted once in the training set and not again in the validation set (ie, with no additional information to validate them) are not shown. Of the quinolones and aminoglycosides, only ofloxacin and amikacin have been included as representatives of their class.

To assess their accuracy, we used training-set characterisations to predict phenotypes for an independent validation set of 1552 isolates that included 449 isolates that were phenotypically resistant to at least one drug, 284 that were multidrug resistant and three that were extensively drug resistant (table, figure 2, appendix 1). 58 (48·3%) of 120 mutations characterised as resistance determining in the training set, and 175 (22·7%) of 772 characterised as benign, recurred in validation-set isolates. These mutations predicted 89·2% of validation-set phenotypes as resistant or susceptible with a mean 92·3% sensitivity (95% CI 90·7–93·7) and 98·4% specificity (98·1–98·7), using ofloxacin and amikacin as representatives of their respective drug classes (table). The presence of uncharacterised mutations in validation-set isolates prevented predictions for the remaining 10·8% of phenotypes.

**Table:** Phenotypic predictions for the validation set

	**Phenotypically resistant**						**Phenotypically sensitive**						**All**		**Excluding uncharacterised**		**Uncharacterised**
	**Genotype**					**Total**	**Genotype**					**Total**	**Sensitivity**	**Specificity**	**Sensitivity**	**Specificity**	
	R	R_x_	S_0_	S_B_	U		R	R_x_	S_0_	S_B_	U						
Isoniazid	305	5	18	1	35	364	19	0	1065	52	52	1188	85·2 (81·1–88·7)	98·4 (97·5–99·0)	94·2 (91·1–96·5)	98·3 (97·4–99·0)	5·6%
Rifampicin	263	12	8	1	16	300	9	1	1200	4	38	1252	91·7 (87·9–94·5)	99·2 (98·5–99·6)	96·8 (94·1–98·5)	99·2 (98·5–99·6)	3·5%
Ethambutol	152	6	7	1	26	192	62	5	1003	79	210	1359	82·3 (76·1–87·4)	95·1 (93·8–96·2)	95·2 (90·7–97·9)	94·2 (92·7–95·4)	15·2%
Pyrazinamide	31	12	27	5	104	179	2	0	1218	67	83	1370	24·0 (17·9–30·9)	99·9 (95·5–100·0)	57·3 (45·3–68·7)	99·8 (99·4–100·0)	12·1%
Streptomycin	278	6	6	9	49	348	10	1	970	34	189	1204	81·6 (77·1–85·5)	99·1 (98·4–99·5)	95·0 (91·9–97·2)	98·9 (98·1–99·4)	15·3%
Ofloxacin	2	3	4	2	0	11	0	0	489	134	38	661	45·5 (16·7–76·6)	100·0 (99·4–100·0)	45·5 (16·7–76·6)	100·0 (99·4–100·0)	5·7%
Amikacin	36	16	5	0	2	59	1	2	427	38	140	608	88·1 (77·1–95·1)	99·5 (98·6–99·9)	91·2 (80·7–97·1)	99·4 (98·1–99·9)	21·3%
Total	1067	60	75	19	232	1453	103	9	6372	408	750	7642	77·6 (75·3–79·7)	98·5 (98·2–98·8)	92·3 (90·7–93·7)	98·4 (98·1–98·7)	10·8%

Total sensitivity and specificity data are weighted means (95% CIs). We investigated each drug separately by comparing the phenotype for each across isolates with this data available. The unit of analysis was therefore not an isolate, but a phenotype. R=resistance-determining mutation. R_x_ =resistance determinant only as a mixed base call (heteroresistance). S_0_=zero mutations present. S_B_=only benign mutations present. U=uncharacterised mutations present in the absence of a resistance-determining mutation. Characterised mutations only exclude the U columns. To avoid double counting for several drugs from the same class, ofloxacin and amikacin were included as representatives of their antibiotic classes, because these had the most resistant phenotypes. Results for ciprofloxacin, moxifloxacin, kanamycin, and capreomycin are in the appendix.

58 recurring resistance-determining mutations occurred across the resistant and susceptible validation-set phenotypes (distribution listed in appendix 1). 54 (93·1%), including 12 not described in the scientific literature, accurately predicted at least one resistant validation-set phenotype. The proportion of phenotypes accurately predicted resistant varied substantially by drug (table). Predictions for pyrazinamide were the least sensitive. Of 34 *pncA* mutations characterised in the training set as resistance determinants, 12 recurred in the validation set. Although 43 (97·7%) of 44 validation-set isolates in which these mutations were noted were phenotypically resistant, these accounted for only 24% of pyrazinamide-resistant isolates in the validation set. Conversely, predictions for rifampicin were the most sensitive. 12 (66·7%) of 18 training-set characterised resistance determinants recurred in the validation set, successfully predicting phenotypic resistance in 275 (96·5%) of 285 isolates in which these were noted, and accounting for 91·7% of rifampicin-resistant isolates in the validation set.

Of 1221 resistant phenotypes in the validation set, 94 (7·7%) were incorrectly predicted susceptible. 20 (21·3%) of 94 were due to mutations characterised as benign in the training set, and 74 (78·7%) of 94 had no relevant mutations, suggesting either a phenotypic or labelling error, or a resistance mechanism outside candidate genes. Of 6892 susceptible validation-set phenotypes, 112 (1·6%) were wrongly predicted resistant, 55 (49·1%) of 112 contained mutations at *embB* M306, suggesting associated phenotypic variability. Eight (7·1%), however, contained *katG* S315T, which is more likely to represent a labelling error rather than a phenotypic error (appendix 1).^2^ To assess whether these results depended on the allocation of isolates to training and validation sets, we repeated the algorithm using the validation set as the training set, and vice versa, predicting 93·1% of phenotypes with mean 92·1% (95% CI 90·1–93·7) sensitivity and 97·9% (97·6–98·3) specificity. We did a further 100 iterations of the algorithm, on each occasion randomly allocating samples to training or validation sets. Over these 100 iterations, the mean proportion of predictable phenotypes in validation sets was 92·7%, and the mean sensitivity and specificity 92·4% (means are the same value as the medians; IQR 91·9–93·0) and 98·2% (98·1–98·3), respectively (appendix 1).

In view of the consistency of these results, we compared the original predictions for the validation set with predictions based on mutations probed by three line-probe assays. With the exception of pyrazinamide, for which no line-probe assay exists, these assays collectively predicted validation-set phenotypes with mean 81·6% (95% CI 79·4–83·7) sensitivity and 98·0% (97·6–98·3) specificity, compared with 85·1% (83·0–87·0) and 98·2% (97·9–98·6), respectively, for the algorithmically characterised mutations based on whole-genome sequencing. However, unlike the line-probe assays, we could use the algorithmically identified mutations to unambiguously distinguish between benign and uncharacterised mutations, allowing further improvement to the results by restricting predictions to the 89·2% of predictable validation-set phenotypes. For these, the mean sensitivity and specificity, excluding pyrazinamide, were 94·6% (93·1–95·8) and 98·0% (97·6–98·4), respectively (appendix 1).

**Figure 3:**
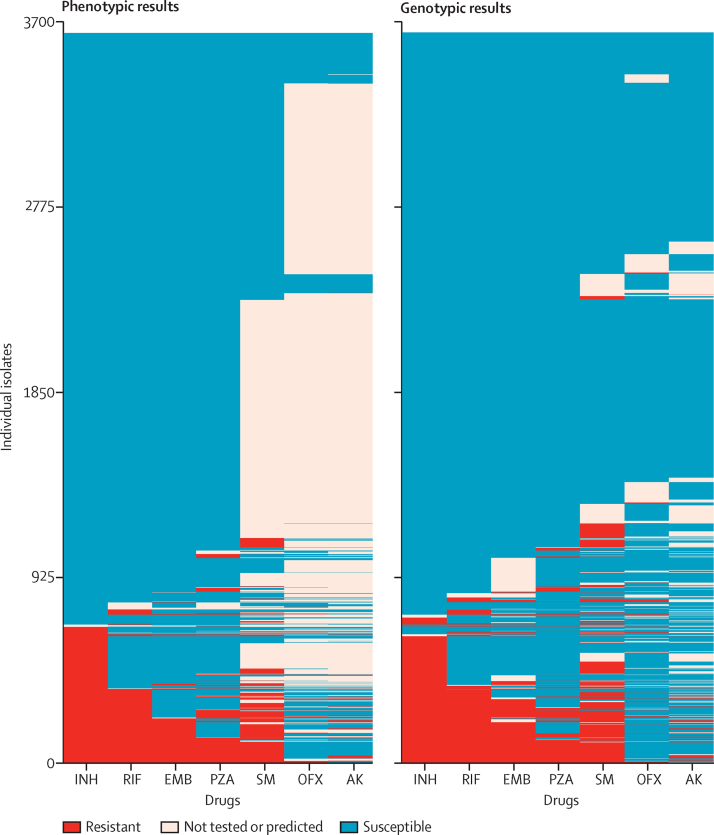
Phenotypic and genotypic antibiograms for all 3651 isolates The left-hand panel shows the phenotypes for seven drugs for the 3651 isolates. The right-hand panel shows the genotypic predictions based on the mutations characterised after applying the algorithm to all 3651 isolates. INH=isoniazid. RIF=rifampicin. EMB=ethambutol. PZA=pyrazinamide. SM=streptomycin. OFX=ofloxacin. AK=amikacin.

The algorithm was rerun for all 3651 isolates, which increased the number of mutations characterised as resistance determining from 120 to 232, and as benign from 772 to 1634. Among the resistance-determining mutations were three that had remained uncharacterised in the original training set, and 16 originally characterised as benign but recharacterised because of additional samples from the phenotypically resistant validation set containing only those mutations. Eight (42·1%) of these 19 mutations had been previously described as resistance determining in the scientific literature (appendix 1). Because all samples were included in this training set, no independent validation set remained, but predictions were made for the entire set itself. 96·1% of phenotypes could be predicted with mean 94·8% (95% CI 93·8–95·7) sensitivity and 98·0% specificity (97·7–98·2; figure 3, appendix 1).

**Figure 4:**
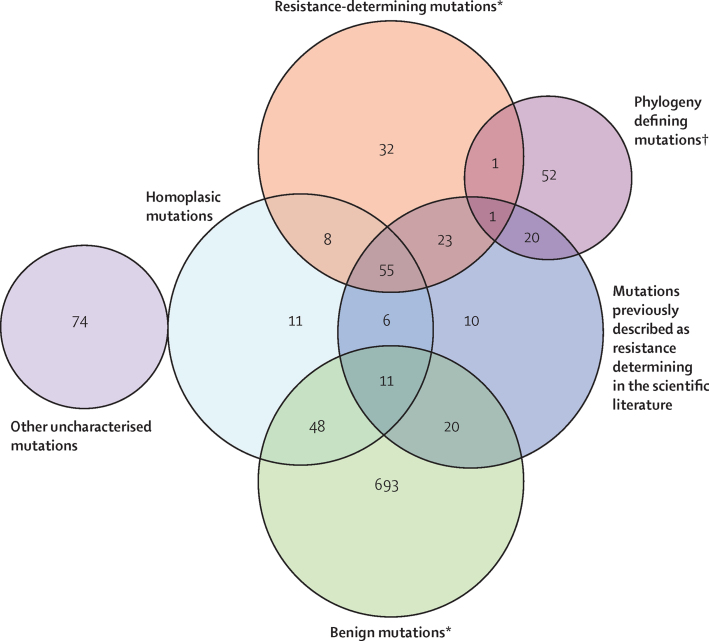
Training-set-characterised mutations Numbers represent the number of mutations for each characterisation. *Among resistance determinants and benign mutations, 15 and 55 insertions and deletions, and 25 and 371 mutations seen in only one isolate respectively, were not or could not be assessed for homoplasy. †*gyrA* A384V defines the Indian Ocean lineage (all isolates in the lineage have this single-nucleotide polymorphism) but is also in one European American isolate. *rpsA* A440T defines *Mycobacterium bovis* but is also in one Central Asian isolate. Both are thereby homoplasic.

We assessed all nucleotide positions across the phylogeny of all 3651 isolate samples for homoplasy to explore first whether characterised resistance determinants were under selection pressure, and then to identify which mutations beyond the 23 candidate genes might be similarly under selection pressure, and therefore plausibly resistance determining. Across the 23 concatenated candidate-gene sequences, 292 (0·8%) of 38 257 nucleotide positions were homoplasic. These affected 63 (52·5%) of 120 resistance determinants, 17 (16·8%) of 101 uncharacterised mutations, and 59 (7·6%) of 772 benign mutations, as characterised in the training set (figure 4). Outside the 23 candidate genes, 5427 (0·1%) of 4 373 275 nucleotide positions were homoplasic, involving 2341 (59·3%) of 3951 remaining genes in the genome.

To increase the probability of finding resistance-determining mutations within this many genes, we identified the most homoplasic by summing the maximum number of homoplasic emergences affecting mutations across each gene. For ten of 14 genes providing the 120 resistance determinants in the training set there were a median 102 emergences (IQR 32–1070), placing them among the 34 (1·4%) of 2364 most homoplasic genes. This compared with a median of five emergences (two to ten) for the other four of 14 genes, and four emergences (two to six) for the remaining genes in the genome.

We searched the 34 most homoplasic genes for non-synonymous mutations that might account for any of the 33 unaccounted-for resistant phenotypes in the training set. These mutations were however predominantly associated with susceptible phenotypes in other isolates (median 12·5% phenotypically resistant, IQR 9–28%) (appendix 1). Those most strongly associated with phenotypic resistance were *rpoC* G332R for ethambutol (five of six resistant), and *phoR* P186L (three of five) for isoniazid. However, all six isolates containing *rpoC* G332R were phenotypically resistant to rifampicin (all contained *rpoB* S450L), suggesting that *rpoC G*332R was more likely to be a compensatory mutation for rifampicin, than the cause of ethambutol resistance. The two other isoniazid-resistant isolates containing *phoR* P186L both also contained the derived resistance determinant *fabG*1 G-17T. No additional convincing resistance determinants were therefore noted.

## Discussion

We used a training set of 2099 *M tuberculosis* genomes to algorithmically characterise mutations across 23 candidate genes as either resistance determining or benign. These characterised mutations predicted 89·2% of phenotypes for an independent validation set of 1552 isolates with high sensitivity and specificity of 92·3% (95% CI 90·7–93·7) and 98·4% (98·1–98·7).

84% of susceptible phenotypes contained no relevant mutations compared with the pan-susceptible reference, and 74% of resistant phenotypes contained exactly one—these findings were key to the characterisation of mutations. Phenotypes were successfully predicted because the same resistance determinants happened repeatedly, independently across isolates. Results were therefore largely independent of training and validation-set composition.

The characterisation of all mutations offers advantages over line-probe assays and other commercial molecular assays. First, data from whole-genome sequencing can be screened for all resistance determinants, resulting in a higher sensitivity than for the mutations based on line-probe assays alone.^21^, ^22^ Second, although line-probe assays can suggest which drugs to avoid by screening a few key resistance-determining mutations, they leave some doubt about which drugs to give. By characterising mutations as benign, we can actively predict phenotypic susceptibility in some isolates, contrasting them from others containing uncharacterised mutations. Third, drug-susceptibility testing based on whole-genome sequencing can be done for additional and even novel drugs at no additional cost, contingent only on the knowledge base of characterised mutations. This wide application could be helpful when designing new treatment regimens.^23^, ^24^

Despite the success of the algorithm, some mutations could not be correctly characterised, and some resistant phenotypes could not be ascribed a causative mutation. One reason is imperfect phenotypic drug-susceptibility testing,^25^, ^26^ best shown by the weak association between *embB* M306I and ethambutol resistance noted both within and across study sites.^20^ Since the algorithm implicitly upweights single recordings of resistance over susceptibility, new samples could lead to the recharacterisation of mutations from benign to resistant, although rarely vice versa (appendix 1). Nevertheless, because whole-genome sequencing variant calling is highly reproducible,^10^, ^27^ phenotypic variability around some mutations will become apparent in large datasets, including those with more resistance to second-line drugs, for which predictions could be recast within a Bayesian analysis framework.^28^ The reproducibility and robustness of sequencing data also has the potential for in-vitro phenotypic techniques to be recalibrated and reassessed. However, if further evidence for the effect of particular mutations is warranted, additional approaches such as in-vitro mutagenesis or crystallographic protein analysis might still be needed.^8^, ^29^

Another possible reason is the presence of mechanisms of resistance outside candidate genes. The homoplasic signal of selection pressure has previously been used by Farhat and colleagues to associate genome-wide mutations with resistance.^8^ The *ponA1* mutations they associated with rifampicin resistance were, however, not homoplasic in these data, and the only non-synonymous *ponA1* mutation that was homoplasic (*ponA1* D24N) was only present in five isolates susceptible to rifampicin. Overall, in this much larger dataset we were not able to identify further resistance determinants associated with homoplasic nucleotide positions outside candidate genes.

**Figure 5:**
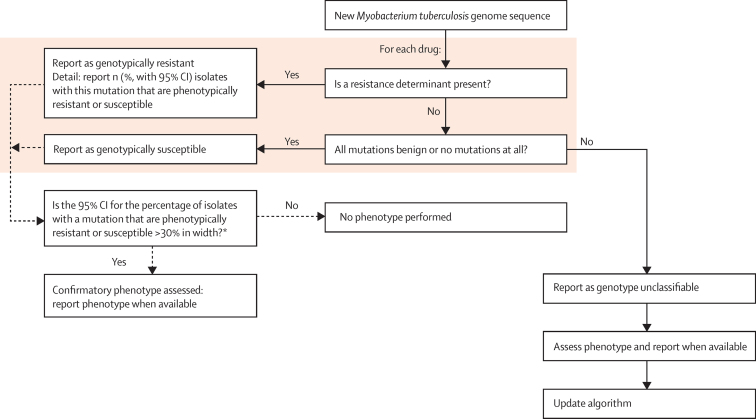
Proposed workflow for transition towards whole-genome sequencing-based drug-susceptibility testing *The 30% CI width suggested is arbitrary, and represents how the precise proportion of isolates with a mutation is probably less relevant than understanding whether this proportion is very high, moderate, or low. However, the precise width could be determined by what is regarded as an acceptable degree of clinical risk, and could also vary by the estimate of proportion resistant. For example, with a targeting width of less than 30%, ten phenotypically resistant isolates of ten isolates with a mutation (100%) has a lower 97·5% CI of 69%, so mutations that are uniformly resistant would need to be phenotyped 11 times before confirmatory phenotyping would stop. For a mutation associated with resistance in 50% of isolates, phenotyping would need to happen 48 times, and for a mutation associated with resistance in either 25% or 75% isolates, 36 times.

Figure 5 suggests one approach to integrating the algorithm into a routine laboratory workflow. Here, phenotypic predictions based on whole-genome sequencing would be made for isolates containing resistance determinants, only benign mutations, or no relevant mutations. As data accrue, confidence in the characterisation of each mutation will grow to the point where routine phenotyping can be restricted to isolates containing uncharacterised mutations that prevent phenotypic prediction. Phenotyping is likely to persist longer for some drugs than for others: although *katG* S315T and *rpoB* S450L were the dominant mutations for isoniazid and rifampicin, we identified many infrequently occurring resistance-determining mutations in *pncA*, as also shown in another study.^30^

Limitations to this study include the few isolates that are phenotypically resistant to second-line drugs. Nevertheless, we show that the algorithm can be successfully applied across the full range of drugs studied, and that the characterisation of mutations as benign is as important as the characterisation of resistance determinants. A further limitation is that we did not have the resources to systematically rephenotype and resequence discordant isolates. However, the size of the study does allow the penetrance of mutations to be assessed across many isolates, mitigating the effects of phenotypic error. Moreover, because the presence of the high-level resistance determinant *katG* S315T in isoniazid-susceptible isolates has been proposed as a marker of sample mislabelling, our rate of nine (1·9%) of 480 discordant isolates compares favourably with previous reports.^2^ Nevertheless, this limitation increases the difficulty of assessing the importance of drug-resistance determinants outside the 23 candidate genes versus phenotypic error or mislabelling to our false-susceptible predictions. Finally, the training and validation sets were drawn from different populations as a consequence of availability at different times. However, our simulation study showed the robustness of our approach.

Public Health England has started to do whole-genome sequencing in parallel to workflows to assess its suitability as a one-stop diagnostic platform for mycobacterial infections. Parallel phenotypic drug-susceptibility testing will lend support to the status of some mutations, and characterise further ones. The cosmopolitan nature of tuberculosis in the UK will enhance our understanding of molecular determinants of resistance, as will the global accumulation of data from whole-genome sequencing. Rollout of the GeneXpert diagnostic test for *M tuberculosis* DNA and resistance to rifampicin has set a precedent for the deployment of advanced technology in low-income, high-burden settings;^31^ with cost-effective, field-ready, sequencing platforms such as the Oxford Nanopore MinION on the horizon,^32^ the prospect of delivering drug-susceptibility testing based on whole-genome sequencing globally, including to settings where no phenotypic drug-susceptibility testing currently exists, is a possibility. Outbreaks could be detected from the same data at no additional cost, potentially adding to local tuberculosis control.^11^ Advances in software now enable sequence data analysis and interpretation to take place without the need for skills in bioinformatics,^33^, ^34^ removing this obstacle to rollout. As techniques to extract genomic DNA for whole-genome sequencing from primary samples improve,^35^ the main remaining scientific challenge to the success of drug-susceptibility testing based on whole-genome sequencing will therefore be the composition of a comprehensive catalogue of characterised mutations.

For the **Tuberculosis Drug Resistance Mutation Database** see https://tbdreamdb.ki.se
