# Corrections

**DOI:** 10.1016/S1473-3099(17)30688-6

**Published:** 2018-01

*Walker TM, Kohl TA, Omar SV, et al. Whole-genome sequencing for prediction of Mycobacterium tuberculosis drug susceptibility and resistance: a retrospective cohort study.* Lancet Infect Dis *2015; **15**: 1193–202*—In the appendix to this article, the genome insertions and deletions (‘indels’) were wrongly annotated. The correct annotations have been added to the online excel appendix as of Nov 21, 2017. Instructions on how to read the new indels are below.

The annotations have each been matched to the corresponding original annotations for comparison. Genome positions corresponding to the NC_000962.2 reference genome are also given. If an insertion or deletion extends into a gene or the 100 base pairs upstream of it, it was recorded and annotated as follows: Gene + nucleotide position in gene + insertion or deletion + nucleotide sequence inserted or deleted. Where there is a sequence deleted as well as inserted, the gene position is given along with the length of the deletion (so it can be retraced) and the sequence corresponding to the insertion. Gene positions and corresponding reference nucleotides are given for the positive strand in all cases and are hence to be read from left to right on that positive strand. For example, for a gene read in the positive direction: If the reference nucleotide is C at position 10 in the gene and the isolate is reported to have nucleotides CT at that position, then the indel is recorded as being an insertion at position 11 (eg, gene_position11_insT). Likewise, if the reference nucleotides are GC starting at positions 5 in the gene and the isolate is reported to have just a G at those positions, then the indel is reported as a deletion of C at position 6 (e.g. gene_position6_delC). For genes read in the negative direction, the two examples given would be annotated as gene_position9_insT and gene_position4_delC (still reading the nucleotide sequence on the positive strand from left to right). There is one new insertion as a result of the re-annotation as this was previously placed outside of the genes of interest. This is in isolate SAMN03647814, affecting gene iniA. This isolate was phenotypically susceptible and the change in annotation has no impact on any of the results presented in the paper. There are four insertions that are now no longer included. All were upstream to the pncA gene but the corrected annotation places them outside of the 100 upstream base pairs explored in the study. These affected isolates SAMN03648036, SAMN03648187, SAMN03648251, and SAMN03648290. All isolates were phenotypically susceptible to pyrazinamide and none of these re-annotations have an impact on any of the results presented in the paper.

