# Supplementary material for: Whole-genome sequencing for prediction of Mycobacterium tuberculosis drug susceptibility and resistance: a retrospective cohort study
Source: Lancet Infect Dis. 2015 Oct;15(10):1193–202. doi: 10.1016/S1473-3099(15)00062-6 (PMC4579482; doi:10.1016/S1473-3099(15)00062-6)
Supplement: Supplementary appendix [file mmc1.pdf]

THE LANCET Infectious Diseases

Supplementary webappendix 1

This webappendix formed part of the original submission and has been peer reviewed. We post it as supplied by the authors.

Supplement to: Walker TM, Kohl TA, Omar SV, et al. Whole-genome sequencing for prediction of Mycobacterium tuberculosis drug susceptibility and resistance: a retrospective cohort study. *Lancet Infect Dis* 2015; published online June 24. http://dx.doi.org/10.1016/S1473-3099(15)00062-6.

Supplementary material

Table of Contents

S1 **2**
S2 **3**
S3 **3**
S4 **5**
S5 **8**
S6 **10**
S6.1 12
S7 **14**
S8 **15**
S8.1 18
S9 **22**
S10 **24**
S11 **26**
S12 **29**
S13 **31**
S14 **32**
S15 **37**
S16 **39**
S17 **40**
References **41**

S1

Samples were selected from different and cosmopolitan sources, ensuring representation of all global clades seen here in a maximum likelihood tree of all 3651 samples. The tree was built from full genome sequences for which nucleotide null-calls at invariant sites were substituted with the reference nucleotide.

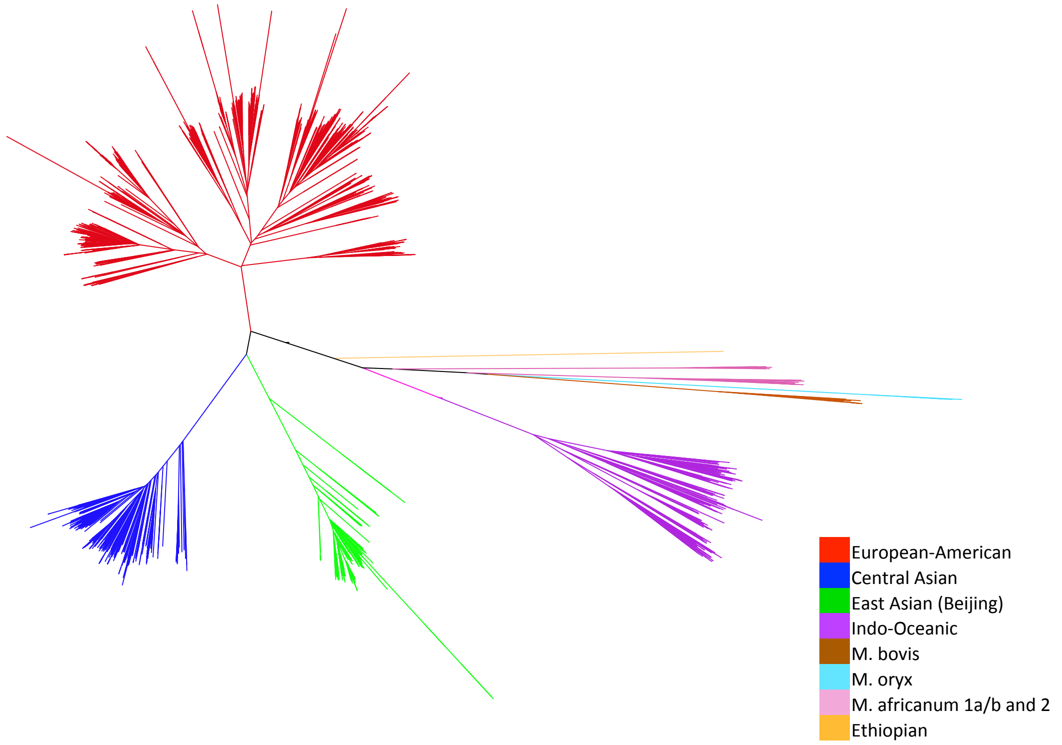

For the original training-set, all isolates sequenced for population studies of Birmingham (2009-2013, unpublished) and Oxfordshire (2007-2012)[1] were selected. Samples sequenced for a study on tuberculosis transmission[2] were also selected, as were all samples phenotypically resistant to isoniazid, or mono-resistant to rifampicin, and stored at the Public Health Laboratory for the Midlands. Samples from Sierra Leone were selected from a study of re-treated smear-positive cases in Kenema district, 2003-2004.[3] Samples from South Africa were sequenced as part of a survey of drug resistance in the country. All phenotypically resistant isolates that were available at the time of asking were contributed first to the training-set and then at a later date to the validation-set, in each case with matched susceptible controls. For the original validation-set, samples were additionally obtained from a population study of Hamburg, Germany (2005-2012) and from a drug-resistance survey of Nukus, Uzbekistan (2001-2006).

NB: The tree gave rise to a small number of unexpectedly long branches. On closer examination this was identified as a consequence of missing data and / or contamination affecting the 16s and 23s genes in a small number of samples. The analysis was re-run without these samples without any material difference to the results.

S2

Sample selection and penotyping. Samples were excluded if they had either no available phenotypic data or where less than 88% of nucleotides in the reference genome were called.

Training-set	Identified	Included
Midlands, UK (unselected)	1162	1122
Midlands, UK (outbreaks)	428	412
Midlands , UK (resistance)	95	94
Oxfordshire, UK (unselected)	389	338
Gauteng province, South Africa	80	54
Kenema district, Sierra Leone	80	79
Total	2234	2099

Validation-set	Identified	Included
Hamburg, Germany	942	841
Nukus, Uzbekistan	277	261
Gauteng province, South Africa	466	450
Total	1685	1552

Phenotypic drug-susceptibility testing was performed at different mycobacterial reference laboratories. UK samples from the Midlands underwent phenotypic testing at the Public Health Laboratory for the Midlands in Birmingham, and Oxfordshire samples were tested at the National Mycobacterial Reference Laboratory in London, all as part of routine patient care. Samples from Sierra Leone, Uzbekistan and Germany were tested at the Forschungszentrum Borstel, Germany, and samples from South Africa at the National Institute for Communicable Diseases, Johannesburg, all as part of research projects.

S3

The 23 candidate-genes were identified in a review of the published literature by SF and SN, who used their expert opinion / judgement to compile a list of high-confidence resistance-determining mutations. This was done prior and hence independent of this study. These were nevertheless taken as a starting point for our study. Genes are listed below together with the unique Pubmed identifiers for the source papers. Each gene was included on the basis of at least one plausible resistance-determining mutation. The publications linked to the pubmed identifiers are listed below the table.

**Genes**	**Pubmed uid**							
aphC	22646308	12654653						
eis	21300839							
embA	20427375	10639358						
embB	20427375	10639358	21300839	9257740	11854934	16641474	22646308	
embC	20427375							
embR	10639358							
fabG1	21300839	19494067						
gidB	22646308							
gyrA	19687244	21300839	21562102					
gyrB	19470506	17412727	19721073					
inhA	21110864							
iniA	10639358							
iniC	10639358							
katG	9210694	21300839	22646308					

manB	10639358							
ndh	11408244							
pncA	22646308	21300839	16848344	15616332	11641519	10681313	9692180	9056006
	9055989	8640557						
rmlD	10639358							
rpoB	11136757	7759399	8027320	8913484	9003625	10565894	10921994	14729930
	15184414	15814606	16229229	16672384	19721079	21300839	22646308	
rpsA	21835980							
rpsL	8849220	22646308						
rrs	21300839	21562102						
tlyA	16048924							

7759399 Bodmer et al, Mutation position and type of substitution in the beta-subunit of the RNA polymerase influence in-vitro activity of rifamycins in rifampicin-resistant Mycobacterium tuberculosis. J Antimicrob Chemother. 1995 Feb;35(2):345-8.

8027320 Kapur et al, Characterization by automated DNA sequencing of mutations in the gene (rpoB) encoding the RNA polymerase beta subunit in rifampin-resistant Mycobacterium tuberculosis strains from New York City and Texas, J Clin Microbiol. 1994 Apr;32(4):1095-8.

8640557 Scorpio et al, Mutations in pncA, a gene encoding pyrazinamidase/nicotinamidase, cause resistance to the antituberculous drug pyrazinamide in tubercle bacillus, Nat Med. 1996 Jun;2(6):662-7.

8849220 Sreevatsan et al, Characterization of rpsL and rrs mutations in streptomycin-resistant Mycobacterium tuberculosis isolates from diverse geographic localities. Antimicrob Agents Chemother. 1996 Apr;40(4):1024-6.

8913484 Moghazeh et al, Comparative antimycobacterial activities of rifampin, rifapentine, and KRM-1648 against a collection of rifampin-resistant Mycobacterium tuberculosis isolates with known rpoB mutations. Antimicrob Agents Chemother. 1996 Nov;40(11):2655-7.

9003625 Kim et al, Mutations in the rpoB gene of Mycobacterium tuberculosis that interfere with PCR-single-strand conformation polymorphism analysis for rifampin susceptibility testing. J Clin Microbiol. 1997 Feb;35(2):492-4.

9055989 Scorpio et al, Characterization of pncA mutations in pyrazinamide-resistant Mycobacterium tuberculosis. Antimicrob Agents Chemother. 1997 Mar;41(3):540-3.

9056006 Sreevatsan et al, Mutations associated with pyrazinamide resistance in pncA of Mycobacterium tuberculosis complex organisms. Antimicrob Agents Chemother. 1997 Mar;41(3):636-40.

9210694 Haas et al, Molecular analysis of katG gene mutations in strains of Mycobacterium tuberculosis complex from Africa. Antimicrob Agents Chemother. 1997 Jul;41(7):1601-3.

9257740 Sreevatsan et al, Ethambutol resistance in Mycobacterium tuberculosis: critical role of embB mutations. Antimicrob Agents Chemother. 1997 Aug;41(8):1677-81

9692180 Hirano et al, Mutation in pncA is a major mechanism of pyrazinamide resistance in Mycobacterium tuberculosis. Tuber Lung Dis. 1997;78(2):117-22.

10565894 Yuen et al, Bacteriological and molecular analysis of rifampin-resistant Mycobacterium tuberculosis strains isolated in Australia. J Clin Microbiol. 1999 Dec;37(12):3844-50.

10639358 Ramaswamy et al, Molecular genetic analysis of nucleotide polymorphisms associated with ethambutol resistance in human isolates of Mycobacterium tuberculosis. Antimicrob Agents Chemother. 2000 Feb;44(2):326-36.

10681313 Cheng et al, pncA mutations as a major mechanism of pyrazinamide resistance in Mycobacterium tuberculosis: spread of a monoresistant strain in Quebec, Canada. Antimicrob Agents Chemother. 2000 Mar;44(3):528-32.

10921994 Valim et al, Mutations in the rpoB gene of multidrug-resistant Mycobacterium tuberculosis isolates from Brazil. J Clin Microbiol. 2000 Aug;38(8):3119-22.

11136757 Heep et al, Frequency of rpoB mutations inside and outside the cluster I region in rifampin-resistant clinical Mycobacterium tuberculosis isolates. J Clin Microbiol. 2001 Jan;39(1):107-10.

11408244 Lee et al, Novel mutations in ndh in isoniazid-resistant Mycobacterium tuberculosis isolates. Antimicrob Agents Chemother. 2001 Jul;45(7):2157-9.

11641519 Lee et al, Characterization of pncA mutations of pyrazinamide-resistant Mycobacterium tuberculosis in Korea. J Korean Med Sci. 2001 Oct;16(5):537-43.

11854934 Lee et al, Mutations in the embB locus among Korean clinical isolates of Mycobacterium tuberculosis resistant to ethambutol. Yonsei Med J. 2002 Feb;43(1):59-64.

12654653 Ramaswamy et al, Single nucleotide polymorphisms in genes associated with isoniazid resistance in Mycobacterium tuberculosisAntimicrob Agents Chemother. 2003 Apr;47(4):1241-50.

14729930 Ramaswamy et al, Genotypic analysis of multidrug-resistant Mycobacterium tuberculosis isolates from Monterrey, Mexico. J Med Microbiol. 2004 Feb;53(Pt 2):107-13.

15184414 Sajduda et al, Molecular characterization of rifampin- and isoniazid-resistant Mycobacterium tuberculosis strains isolated in Poland. J Clin Microbiol. 2004 Jun;42(6):2425-31.

15616332 Rodrigues et al, Characterization of pncA mutations in pyrazinamide-resistant Mycobacterium tuberculosis in Brazil. Antimicrob Agents Chemother. 2005 Jan;49(1):444-6.

15814606 O'Sullivan et al, Analysis of rpoB and pncA mutations in the published literature: an insight into the role of oxidative stress in Mycobacterium tuberculosis evolution? J Antimicrob Chemother. 2005 May;55(5):674-9.

16048924 Maus et al, Molecular analysis of cross-resistance to capreomycin, kanamycin, amikacin, and viomycin in Mycobacterium

	tuberculosis. Antimicrob Agents Chemother. 2005 Aug;49(8):3192-7.
**16229229**	Hillemann et al, Rifampicin and isoniazid resistance mutations in Mycobacterium tuberculosis strains isolated from patients in Kazakhstan. Int J Tuberc Lung Dis. 2005 Oct;9(10):1161-7.
**16641474**	Plinke et al, Significance of mutations in embB codon 306 for prediction of ethambutol resistance in clinical Mycobacterium tuberculosis isolates. Antimicrob Agents Chemother. 2006 May;50(5):1900-2.
**16672384**	Park et al, Comparison of a conventional antimicrobial susceptibility assay to an oligonucleotide chip system for detection of drug resistance in Mycobacterium tuberculosis isolates. J Clin Microbiol. 2006 May;44(5):1619-24.
**16848344**	louw et al, Frequency and implications of pyrazinamide resistance in managing previously treated tuberculosis patients. Int J Tuberc Lung Dis. 2006 Jul;10(7):802-7.
**17412727**	Wang et al, Fluoroquinolone resistance in Mycobacterium tuberculosis isolates: associated genetic mutations and relationship to antimicrobial exposure. J Antimicrob Chemother. 2007 May;59(5):860-5. Epub 2007 Apr 5.
**19470506**	Feuerriegel et al, Sequence analyses of just four genes to detect extensively drug-resistant Mycobacterium tuberculosis strains in multidrug-resistant tuberculosis patients undergoing treatment. Antimicrob Agents Chemother. 2009 Aug;53(8):3353-6. doi: 10.1128/AAC.00050-09. Epub 2009 May 26.
**19687244**	Von Groll et al, Fluoroquinolone resistance in Mycobacterium tuberculosis and mutations in gyrA and gyrB. Antimicrob Agents Chemother. 2009 Oct;53(10):4498-500. doi: 10.1128/AAC.00287-09. Epub 2009 Aug 17.
**19721073**	Duong et al , Beijing genotype of Mycobacterium tuberculosis is significantly associated with high-level fluoroquinolone resistance in Vietnam. Antimicrob Agents Chemother. 2009 Nov;53(11):4835-9. doi: 10.1128/AAC.00541-09. Epub 2009 Aug 31.
19721079	Bahrmand et al, High-level rifampin resistance correlates with multiple mutations in the rpoB gene of pulmonary tuberculosis isolates from the Afghanistan border of Iran. J Clin Microbiol. 2009 Sep;47(9):2744-50. doi: 10.1128/JCM.r00548-09.
**20427375**	Plinke et al, embCAB sequence variation among ethambutol-resistant Mycobacterium tuberculosis isolates without embB306 mutation. J Antimicrob Chemother. 2010 Jul;65(7):1359-67. doi: 10.1093/jac/dkq120. Epub 2010 Apr 28.
**21110864**	Ioerger et al, The non-clonality of drug resistance in Beijing-genotype isolates of Mycobacterium tuberculosis from the Western Cape of South Africa. BMC Genomics. 2010 Nov 26;11:670. doi: 10.1186/1471-2164-11-670
21300839	Campbell et al, Molecular detection of mutations associated with first- and second-line drug resistance compared with conventional drug susceptibility testing of Mycobacterium tuberculosis. Antimicrob Agents Chemother. 2011 May;55(5):2032-41. doi: 10.1128/AAC.01550-10. Epub 2011 Feb 7.
**21300839**	Campbell et al, Molecular detection of mutations associated with first- and second-line drug resistance compared with conventional drug susceptibility testing of Mycobacterium tuberculosis. Antimicrob Agents Chemother. 2011 May;55(5):2032-41. doi: 10.1128/AAC.01550-10. Epub 2011 Feb 7.
**21562102**	Huang et al, Performance assessment of the GenoType MTBDRsl test and DNA sequencing for detection of second-line and ethambutol drug resistance among patients infected with multidrug-resistant Mycobacterium tuberculosis. J Clin Microbiol. 2011 Jul;49(7):2502-8. doi: 10.1128/JCM.00197-11. Epub 2011 May 11.
**21835980**	Shi et al, Pyrazinamide inhibits trans-translation in Mycobacterium tuberculosis. Science. 2011 Sep 16;333(6049):1630-2. doi: 10.1126/science.1208813. Epub 2011 Aug 11.
22646308	Feuerriegel et al, Sequence analysis for detection of first-line drug resistance in Mycobacterium tuberculosis strains from a high-incidence setting. BMC Microbiol. 2012 May 30;12:90. doi: 10.1186/1471-2180-12-90.

S4

100 iterations of the algorithm, each time randomly assigning 1825 sequences to a training-set and 1826 to a validation-set. Genotypic predictions for the phenotypes in each of the 100 validation-sets are shown. As in table 1, algorithmic characterisations are based on: R (resistance-determining mutation); Rx (resistance-determinant as a mixed base-call); S_0 (zero mutations present); Ss (only benign mutations present); U (uncharacterised mutation present in the absence of a resistance-determining mutation).

Phenotypically Resistant					Phenotypically Sensitive						
Genotype					Genotype					Excluding uncharacterised	
R	R_x	S_0	S_s	U	R	R_x	S_0	S_s	U	Sensitivity	Specificity
786	30	53	27	98	143	9	6754	565	591	91·07	97·97
786	37	46	14	95	130	12	6842	504	565	93·20	98·10
877	38	52	14	106	147	9	6754	536	558	93·27	97·90
807	37	56	8	106	129	14	6789	531	540	92·95	98·08
783	42	54	18	92	125	38	6804	487	606	91·97	97·81
776	36	44	12	86	131	12	6780	559	594	93·55	98·09
878	44	50	13	97	135	7	6708	534	535	93·60	98·08
848	46	59	11	88	102	14	6744	546	593	92·74	98·43
817	32	56	17	85	124	10	6783	570	545	92·08	98·21

843	39	59	12	97	128	10	6837	569	503	92·55	98·17
850	40	43	13	92	130	12	6810	510	564	94·08	98·10
835	42	53	18	90	118	10	6746	562	574	92·51	98·28
796	36	50	13	100	115	10	6722	582	558	92·96	98·32
828	38	61	18	88	112	6	6792	552	571	91·64	98·42
884	42	58	15	104	129	9	6762	569	506	92·69	98·15
843	35	59	7	130	134	10	6733	528	628	93·01	98·06
882	39	57	16	106	107	10	6744	531	562	92·66	98·42
883	41	37	12	100	114	12	6791	556	535	94·96	98·31
820	42	58	13	93	139	11	6775	519	595	92·39	97·98
850	32	66	22	109	131	15	6746	590	532	90·93	98·05
829	47	65	18	93	124	15	6738	536	585	91·35	98·12
814	31	59	10	97	146	8	6763	566	568	92·45	97·94
812	38	48	12	92	135	7	6774	544	558	93·41	98·10
836	43	58	10	115	122	8	6743	514	601	92·82	98·24
843	33	50	18	87	144	30	6768	557	517	92·80	97·68
822	37	54	18	126	122	13	6753	605	467	92·27	98·20
836	37	60	14	102	133	8	6837	558	586	92·19	98·13
816	37	53	11	112	112	11	6788	511	617	93·02	98·34
846	38	52	19	98	112	12	6776	582	571	92·57	98·34
794	39	58	22	90	116	13	6722	513	622	91·24	98·25
827	33	59	16	88	124	12	6756	548	534	91·98	98·17
702	29	57	18	95	116	11	6872	542	578	90·69	98·32
859	36	65	20	79	122	10	6705	587	540	91·33	98·22
846	40	58	22	118	134	9	6764	526	573	91·72	98·08
815	43	39	25	97	107	11	6784	526	571	93·06	98·41
776	34	56	17	91	125	11	6826	541	583	91·73	98·19
865	40	58	15	94	133	9	6760	563	548	92·54	98·10
830	40	57	16	112	120	12	6782	518	548	92·26	98·22
805	38	58	12	90	130	9	6858	582	520	92·33	98·17
844	39	49	22	103	133	14	6721	549	556	92·56	98·02
851	41	52	22	96	131	10	6703	536	590	92·34	98·09
815	42	62	10	104	152	9	6797	504	582	92·25	97·84
851	50	63	28	102	133	10	6750	523	584	90·83	98·07
849	39	60	16	104	132	8	6787	536	559	92·12	98·12
928	40	45	16	99	139	8	6685	614	501	94·07	98·03
838	39	51	10	105	112	5	6767	519	581	93·50	98·42
829	38	61	18	85	120	11	6804	514	533	91·65	98·24
829	38	44	16	111	133	11	6714	613	568	93·53	98·07
842	37	56	13	104	121	6	6734	531	600	92·72	98·28
835	48	61	12	106	127	12	6721	558	567	92·36	98·13
811	34	49	19	105	112	9	6827	538	536	92·55	98·38
884	44	48	18	104	118	10	6669	560	615	93·36	98·26
827	35	58	14	79	137	12	6807	514	564	92·29	98·01
860	37	60	16	122	122	8	6774	565	535	92·19	98·26
807	41	65	13	97	146	12	6793	559	559	91·58	97·90

844	45	48	19	104	133	9	6775	551	482	92·99	98·10
839	48	49	15	107	132	11	6729	542	577	93·27	98·07
838	42	48	14	81	139	31	6754	508	565	93·42	97·71
836	47	42	9	95	136	14	6788	581	511	94·54	98·01
872	31	50	17	109	139	5	6779	552	524	93·09	98·07
830	40	47	17	108	141	6	6734	621	524	93·15	98·04
845	36	60	20	107	125	13	6753	596	527	91·68	98·16
844	35	59	21	87	119	11	6791	520	558	91·66	98·25
872	42	57	24	91	128	11	6758	542	557	91·86	98·13
875	35	60	14	88	128	11	6792	512	616	92·48	98·13
782	39	51	14	98	119	6	6742	524	563	92·66	98·31
781	33	65	17	109	123	8	6817	578	554	90·85	98·26
887	41	48	12	105	124	10	6728	551	539	93·93	98·19
823	32	56	12	99	113	13	6779	535	532	92·63	98·31
878	38	57	9	101	118	13	6766	539	535	93·28	98·24
835	32	45	13	94	115	7	6812	518	617	93·73	98·36
836	32	64	22	95	145	8	6767	585	540	90·99	97·96
825	43	60	11	81	140	14	6764	567	537	92·44	97·94
806	39	54	29	106	113	12	6763	594	551	91·06	98·33
844	32	48	11	90	136	9	6799	546	534	93·69	98·06
812	40	55	15	92	126	10	6729	524	617	92·41	98·16
770	37	52	18	96	131	8	6759	517	583	92·02	98·13
808	30	55	19	111	112	8	6792	568	530	91·89	98·40
953	46	57	19	88	134	10	6690	554	554	92·93	98·05
873	39	54	15	108	131	14	6770	547	513	92·97	98·06
847	43	51	22	81	105	8	6771	566	521	92·42	98·48
854	32	58	19	95	121	7	6737	507	592	92·00	98·26
818	36	54	9	103	130	10	6724	553	587	93·13	98·11
811	29	63	17	98	108	10	6788	605	474	91·30	98·43
852	40	57	16	105	117	14	6825	581	514	92·44	98·26
819	49	60	19	87	104	8	6761	595	530	91·66	98·50
832	43	54	17	107	127	7	6735	525	619	92·49	98·19
783	46	48	24	82	113	9	6874	513	583	92·01	98·38
845	32	53	24	112	121	12	6766	531	589	91·93	98·21
800	37	52	12	119	133	6	6786	553	561	92·90	98·14
860	35	50	17	84	136	8	6744	630	515	93·04	98·08
855	35	60	24	99	133	8	6735	570	502	91·38	98·11
832	38	50	24	103	127	10	6718	502	612	92·16	98·14
763	42	54	13	109	141	14	6748	549	545	92·32	97·92
803	41	54	22	105	124	11	6755	541	541	91·74	98·18
849	33	66	17	114	127	8	6719	500	606	91·40	98·16
789	43	55	21	102	123	12	6751	588	499	91·63	98·19
811	36	48	17	102	113	8	6752	527	622	92·87	98·36
837	36	59	14	96	126	10	6777	530	608	92·28	98·17
841	38	57	17	104	129	13	6769	534	534	92·24	98·09

S5

Phylogeny defining SNPs removed by the algorithm: SNPs listed in either Coll *et. al.* *(*25176035[uid]) or Feuerriegel *et. al.* *(*24458512[uid]), that were in the 23 candidate-genes, and that occurred in any of the 3651 sequences, were idenified and set aside by the algorithm. Additional lineage or sub-lineage associated SNPs not listed in the referenced papers were identified manually and also set aside (source left as blank in table, but lineage either given in the table or location of SNP indicated in figure where it defines a sub-lineage).

**Lineage defining mutations**	**SNPs**	**Source**	**Lineage name (spoligotype-based names taken from Coll *et. al.*)**
ahpC_G-88A	G/A	Coll *et. al.* 25176035[uid]	Central Asian
embA_P913S	CCG/TCG	Coll *et. al.* 25176035[uid]	Indo-Oceanic
embA_T608N	ACC/AAC	Coll *et. al.* 25176035[uid]	Euro-American (T2)
embA_V206M	GTG/ATG	Coll *et. al.* 25176035[uid]	Indo-Oceanic (EAI4;EAI5;EAI6;EAI3)
embB_E378A	GAG/GCG	Feuerriegel *et. al.* 24458512[uid]	Indo-Oceanic, Ethiopian, *M. africanum 1* & 2, *M. bovis*
embB_Q139H	CAG/CAC	Coll *et. al.* 25176035[uid]	Euro-American (X-type)
embC_N394D	AAC/GAC	Coll *et. al.* 25176035[uid]	Indo-Oceanic
embC_R567H	CGC/CAC	Coll *et. al.* 25176035[uid]	Central Asian (CAS1-Kili)
embC_R738Q	CGG/CAG	Coll *et. al.* 25176035[uid]	Central Asian
embC_T270I	ACC/ATC	Feuerriegel *et. al.* 24458512[uid]	*M. africanum* and animal lineages
embC_V104M	GTG/ATG		Central Asian sub-lineage
embC_V981L	GTG/CTG	Coll *et. al.* 25176035[uid]	Euro-American (T;H;X)
embR_C110Y	TGC/TAC	Coll *et. al.* 25176035[uid]	Indo-Oceanic
embR_H124R	CAC/CGC	Coll *et. al.* 25176035[uid]	Ethiopian
embR_L313R	CTG/CGG	Coll *et. al.* 25176035[uid]	Euro-American (T2)
embR_Y216H	TAC/CAC	Coll *et. al.* 25176035[uid]	Euro-American (T3)
gidB_A119T	GCC/ACC		Indo-Oceanic sub-lineage
gidB_E92D	GAA/GAC	Coll *et. al.* 25176035[uid]	East Asian (Beijing)
gidB_L16R	CTT/CGT	Coll *et. al.* 25176035[uid]	Euro-American (LAM)
gidB_S100F	TCT/TTT		All other than H37Rv
gyrA_A384V	GCA/GTA	Coll *et. al.* 25176035[uid]	Indo-Oceanic
gyrA_E21Q	GAG/CAG		All other than H37Rv
gyrA_G247S	GGC/AGC	Coll *et. al.* 25176035[uid]	Euro-American (LAM9;T5)
gyrA_G668D	GGC/GAC		All other than Euro-American sub-lineage
gyrA_S95T	AGC/ACC	Feuerriegel *et. al.* 24458512[uid]	H37Rv
gyrA_T80A	ACC/GCG	Coll *et. al.* 25176035[uid]	Euro-American (T2-Uganda;T2)
gyrB_A442S	GCG/TCG		*M. bovis* and *M.africanum* 1 & 2
gyrB_C-48T	C/T		Indo-Oceanic sub-lineage
gyrB_M330I	ATG/ATC		Indo-Oceanic
inhA_V78A	GTG/GCG	Coll *et. al.* 25176035[uid]	*M. africanum* 2
iniA_H481Q	CAT/CAG		Indo-Oceanic, Ethiopian, *M. africanum 1* & 2, *M.bovis*
katG_R463L	CGG/CTG	Coll *et. al.* 25176035[uid]	Euro-American
manB_D152N	GAC/ACC		Central Asian
ndh_G-70T	G/T		*M. africanum* 1
ndh_R284W	CGG/TGG		*M. bovis* sub-lineage
ndh_V18A	GTG/GCG	Coll *et. al.* 25176035[uid]	Euro-American (S; T1)
pncA_H57D	CAC/GAC	Feuerriegel *et. al.* 24458512[uid]	*M. bovis*
rmlD_S257P	TCG/CCG		Indo-Oceanic, Ethiopian, *M. africanum 1* & 2, *M.bovis*
rpoB_C-61T	C/T		Central Asian sub-lineage
rpsA_A440T	GCG/ACG	Feuerriegel *et. al.* 24458512[uid]	*M. caprae* (but also *M. bovis*)
rpsA_T459P	ACC/CCC	Coll *et. al.* 25176035[uid]	Ethiopian
rrs_C492T	C/T	Coll *et. al.* 25176035[uid]	Euro-American (LAM3)

Location of sub-lineage defining SNPs not referenced in the literature.

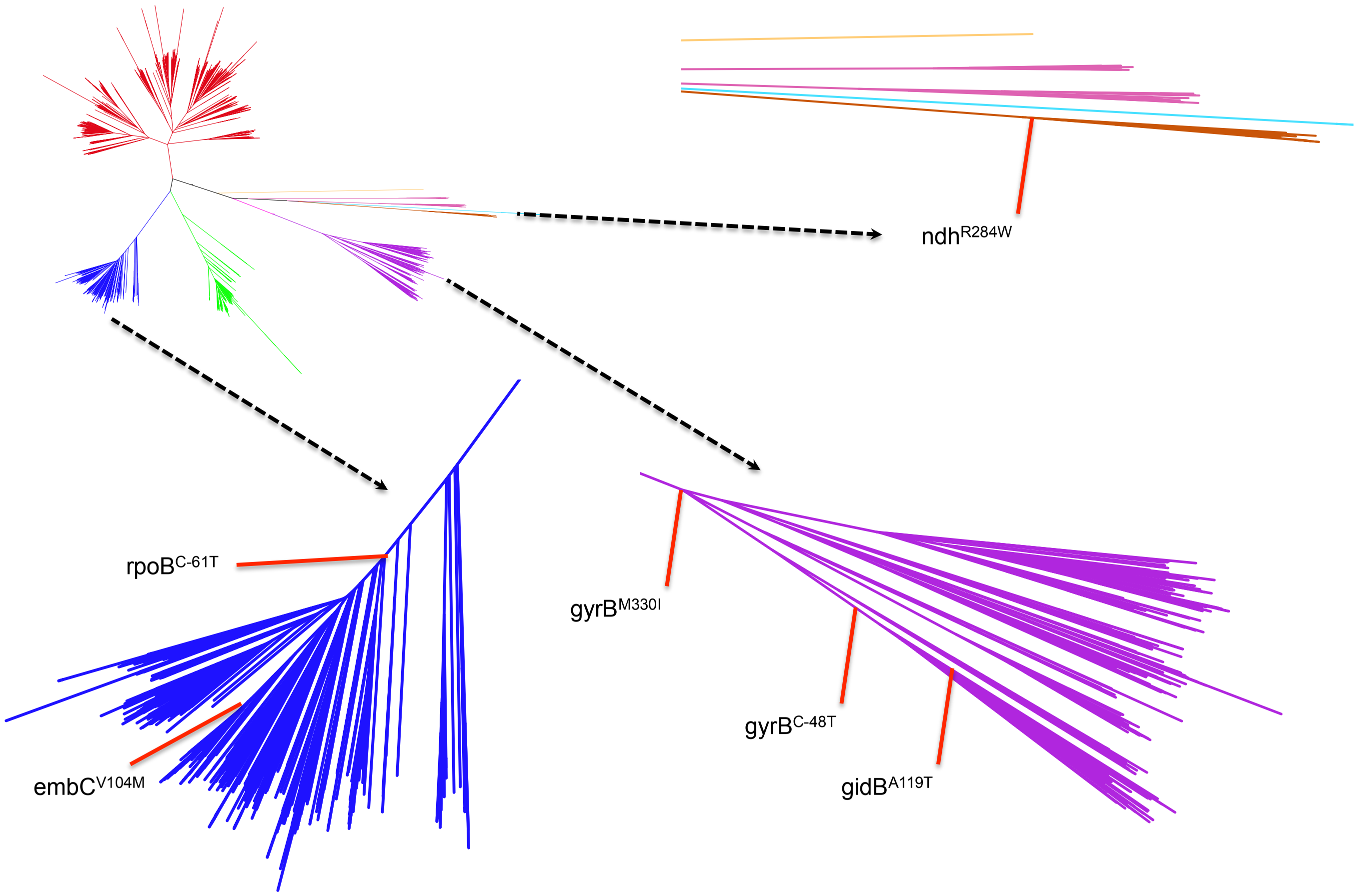

S6

Each step in the algorithm is depicted here as a schematic. First, all sequences were mapped to the H37Rv *M. tuberculosis* reference genome. Across the genes of interest and their promoter regions (defined as 100 base-pairs upstream), all base-calls were identified as either the same as reference, different from reference, or ‘null’ (N) where no base could be called because of insufficient coverage or because there was evidence for more than one base (‘mixed-call’). Five hypothetical sequences are shown here (Seq 1-5).

H37Rv	A	C	G	T	C	C	T	T	G
Seq1	N	C	G	T	C	C	T	T	G
Seq2	A	C	T	T	C	C	T	T	A
Seq3	A	C	G	T	G	C	T	T	G
Seq4	A	C	G	T	C	C	T	T	G
Seq5	A	C	G	T	C	C	T	T	G

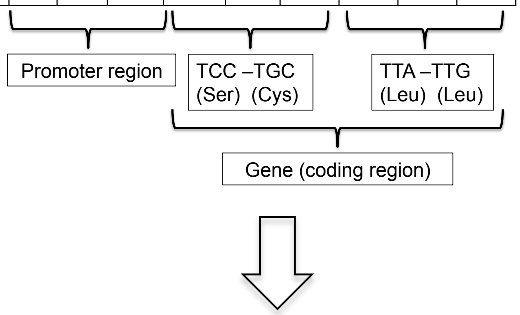

Where one or more sequences differed from the reference just on the basis of a null-call, and all others are identical to the reference, that nucleotide position (be it upstream or within a gene) was set aside and not considered of further interest. Positions at which at least one sequence had a SNP in a promoter region, or a SNP leading to non-synonymous amino-acid substitution, were identified for further analysis. Where codons differed from the reference only on the basis of one or more sequences that had a synonymous SNP within the codon, they were also set aside.

H37Rv	A	C	G	T	C	C	T	T	G
Seq1	N	C	G	T	C	C	T	T	G
Seq2	A	C	T	T	C	C	T	T	A
Seq3	A	C	G	T	G	C	T	T	G
Seq4	A	C	G	T	C	C	T	T	G
Seq5	A	C	G	T	C	C	T	T	G

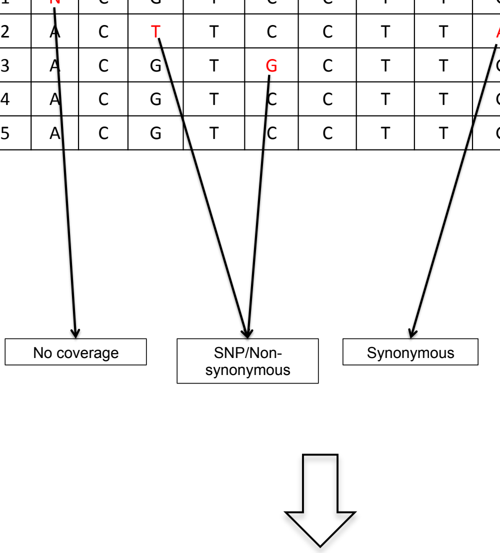

Among the remaining mutations (SNPs, AA substitutions and indels), some were identified as lineage, or phylogeny defining and therefore set aside. Others were only seen in sequences / isolates that had not been phenotyped to the relevant drug. These were also set aside. The remaining mutations were then characterised as either resistance-determining or as benign.

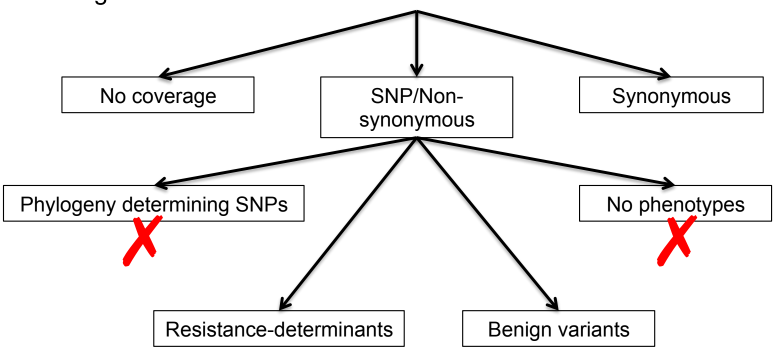

Resistance-determinants were identified by focussing on all genes relevant to a drug. In the example below, the five genes believed to be relevant to isoniazid are shown for a single sample / sequence. Each position within each gene is marked by a coloured line where the reference mutation has not been called. After the null-calls are set aside from otherwise invariant positions, the synonymous mutations are set aside, and the phylogeny/lineage defining mutations ('PhyloSNPs') are set aside, only a single mutation remains (circled). If this sample in question is phenotypically resistant to isoniazid, then this remaining mutation is defined as a resistance-determinant and all other sample containing this mutation (genotypically) predicted to be resistant on this basis.

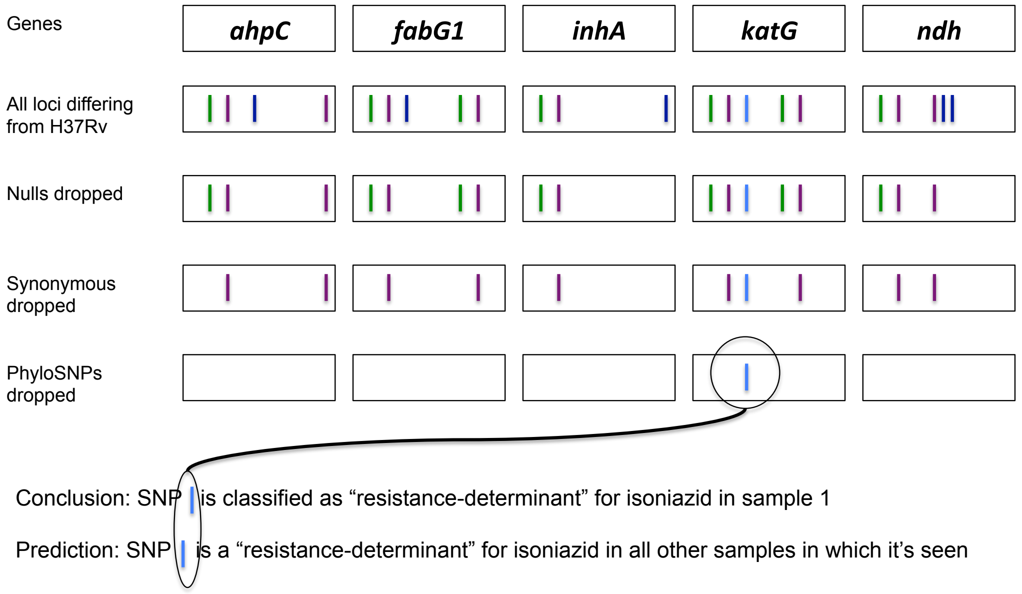

Were the phenotype to have been susceptible in this example, all other isolates containing the circled mutation would have had to have been inspected before the mutation could be characterised. Two scenarios could lead to the mutation being characterised as benign. First, if all isolates containing the mutation were phenotypically susceptible. Second, if all isolates containing only that mutation were phenotypically susceptible (see below).

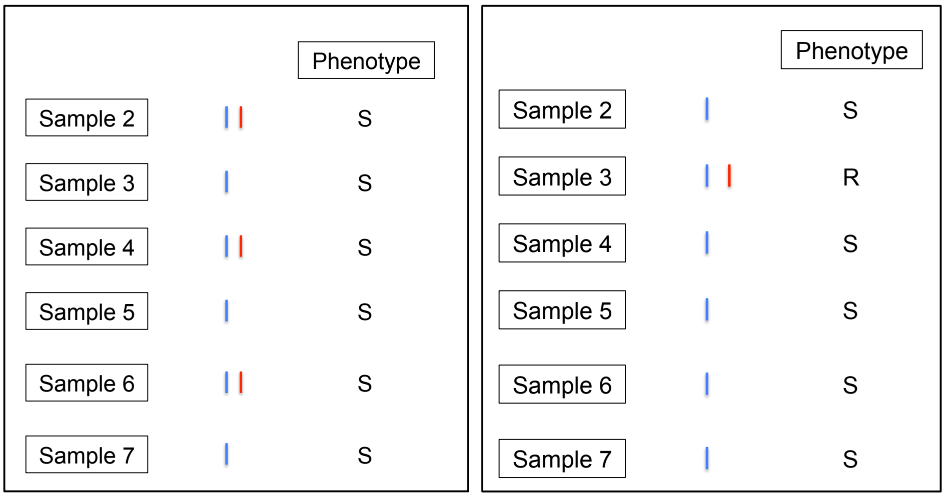

As can be seen in the right hand panel, the benign 'blue mutation' occurs in one phenotypically resistant isolate. Benign mutations were therefore set aside before a second iteration of the algorithm was undertaken in an attempt to reveal additional lone-standing resistance-determinants. The process is illustrated below where the circled mutation characterised as 'benign' on the basis of other samples (as in the right hand panel above) is set aside to reveal a lone-standing mutation that could now be characterised as resistance-determining, in this resistant sample.

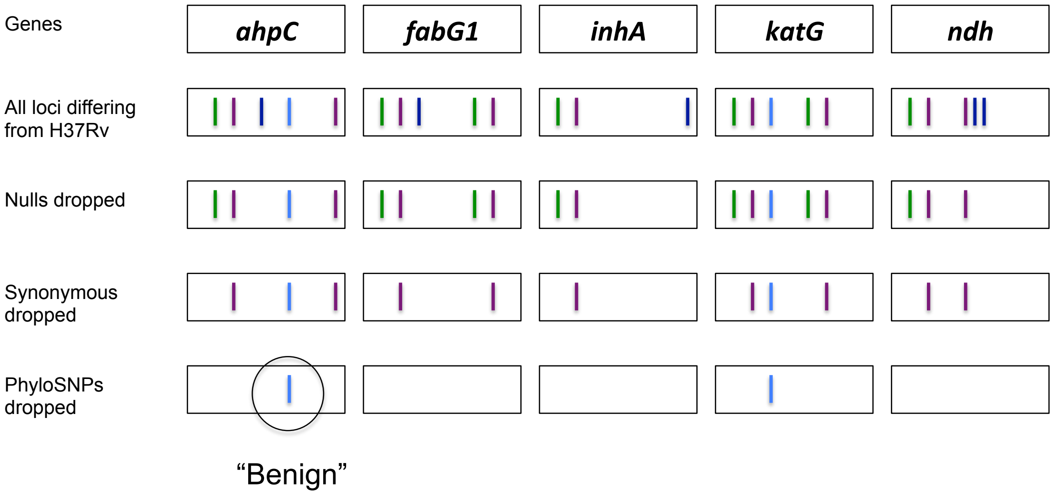

When new sequences are added to a training-set, this has the potential to lead to the re-characterisation of mutations. For example, adding an eighth sample containing the 'blue mutation' to the left hand panel above would lead to the re-characterisation of that mutation as 'resistant' if the phenotype for that eighth sample were resistant. Resistant mutations could however only be re-characterised as benign where their original characterisation as 'resistant' was dependent on another mutation being characterised as benign (as immediately above) *and* where the mutation is otherwise always associated with a susceptible phenotype when occurring as the only mutation in a sample (as in the right hand panel above).

S6.1

The full results of the algorithm as applied to the original training set are shown below.

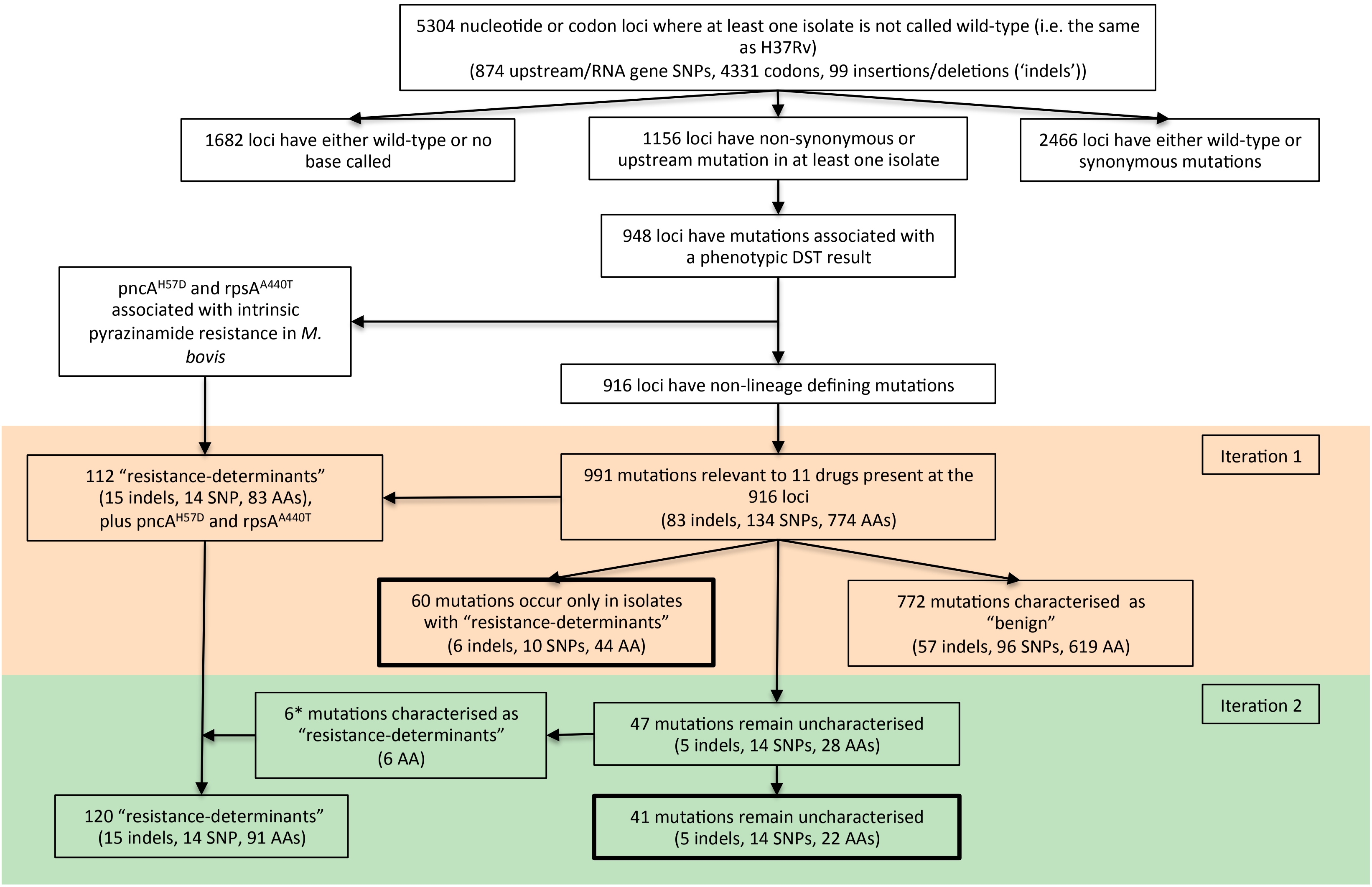
5304 nucleotide or codon loci where at least one isolate is not called wild-type (i.e. the same as H37Rv)
(874 upstream/RNA gene SNPs, 4331 codons, 99 insertions/deletions (‘indels’))
1682 loci have either wild-type or no base called
1156 loci have non-synonymous or upstream mutation in at least one isolate
2466 loci have either wild-type or synonymous mutations
948 loci have mutations associated with a phenotypic DST result
pncA H57D and rpsA A440T associated with intrinsic pyrazinamide resistance in M. bovis
916 loci have non-lineage defining mutations
Iteration 1
112 “resistance-determinants” (15 indels, 14 SNP, 83 AAs), plus pncA H57D and rpsA A440T
991 mutations relevant to 11 drugs present at the 916 loci
(83 indels, 134 SNPs, 774 AAs)
60 mutations occur only in isolates with “resistance-determinants”
(6 indels, 10 SNPs, 44 AA)
772 mutations characterised as “benign”
(57 indels, 96 SNPs, 619 AA)
Iteration 2
6* mutations characterised as “resistance-determinants”
(6 AA)
47 mutations remain uncharacterised
(5 indels, 14 SNPs, 28 AAs)
120 “resistance-determinants”
(15 indels, 14 SNP, 91 AAs)
41 mutations remain uncharacterised
(5 indels, 14 SNPs, 22 AAs)

S7

Uncharacterised mutations that only occur in resistant phenotypes.

Mutations	Drug
ahpC_54_insGT	INH
ahpC_C-54T	INH
ahpC_D73H	INH
ahpC_E76K	INH
ahpC_F108S	INH
ahpC_G-48A	INH
ahpC_G-74A	INH
ahpC_G32D	INH
ahpC_T-42C	INH
ahpC_W96C	INH
fabG1_T-8A	INH
fabG1_T-8G	INH
inhA_I21V	INH
inhA_T162S	INH
katG_24_insCA	INH
katG_A-75G	INH
katG_A109T	INH
katG_A244G	INH
katG_C549S	INH
katG_D542E	INH
katG_G34A	INH
katG_L427F	INH
katG_Q471R	INH
katG_R78G	INH
katG_S211N	INH
katG_T579A	INH
katG_W149R	INH
katG_Y155S	INH
ndh_D143A	INH
ndh_K208E	INH
ndh_K32E	INH
rpoB_389_insATGTCGT	RIF
rpoB_A538V	RIF
rpoB_I480V	RIF
rpoB_P45S	RIF
rpoB_Q975H	RIF
rpoB_R219C	RIF
rpoB_S874F	RIF
rpoB_V168A	RIF
rpoB_V496A	RIF
embA_68_delC	EMB
embA_A813G	EMB
embA_C-11A	EMB

embA_C-8T	EMB
embA_G759R	EMB
embB_G156C	EMB
embB_M423T	EMB
embB_W1089R	EMB
embC_A387V	EMB
embR_458_delG	EMB
iniA_N50D	EMB
iniA_V544A	EMB
iniC_G194R	EMB
iniC_G341V	EMB
gidB_504_delC	SM
tlyA_C-83T	SM
gyrA_D641E	CIP
gidB_L108P	AK
gidB_L108P	CAP
gidB_L108P	KAN

S8

The 120 resistance-determinants derived from the original training-set are shown. Each mutation can be counted more than once if it is characterised as a resistance-determinant to more than one drug. The number of resistant and susceptible phenotypes with which each resistance-determinant is associated is shown, and a Pubmed identifier is given if the mutation has been described as resistance-determining before. 79/120 (66%) resistance-determinant/drug combinations had previously been described in the literature. This compared to 31/772 (4%) 'benign' mutations and 20/71 (28%) mutations defining lineages not intrinsically drug-resistant (counting each mutation once for each drug it was characterised for, i.e. some counting some more than once).

Mutations	Drug	Resistant	Susceptible	Total	Pubmed link to reference previously citing the mutation
ahpC_C-57T	INH	1	0	1	
eis_G-10A	KAN	4	0	4	21300839[uid]
embB_D354A	EMB	1	1	2	20427375[uid]
embB_G406A	EMB	1	3	4	10639358[uid]
embB_G406D	EMB	2	2	4	10639358[uid]
embB_G406S	EMB	3	5	8	10639358[uid]
embB_H1002R	EMB	1	0	1	22646308[uid] **
embB_M306I	EMB	20	34	54	9257740[uid]
embB_M306V	EMB	18	3	21	9257740[uid]
embB_Q497K	EMB	1	2	3	10639358[uid]
embB_Q497R	EMB	8	4	12	10639358[uid]
fabG1_C-15T	INH	73	6	79	10428945[uid]
fabG1_G-17T	INH	5	1	6	14638486[uid]
fabG1_T-8C	INH	3	3	6	15793126[uid]

gidB_141_delC	SM	1	0	1	
gidB_202_delG	SM	4	7	11	
gidB_202_insGC	SM	1	1	2	
gidB_A134E	SM	1	0	1	17238915[uid]
gidB_A138T	SM	1	0	1	
gidB_A138V	SM	2	1	3	
gidB_A200E	SM	2	0	2	17238915[uid]
gidB_A80P	SM	1	0	1	24102832[uid]
gidB_G69D	SM	3	0	3	
gidB_H48N	SM	2	0	2	
gidB_L91P	SM	1	0	1	
gidB_P75L	SM	1	1	2	
gidB_R137W	SM	1	1	2	21444711[uid]
gidB_S70N	SM	1	0	1	
gidB_V65G	SM	1	0	1	
gidB_V88A	SM	2	0	2	
gyrA_A74S	CIP	2	0	2	17035499[uid]
gyrA_A90V	MOX	2	0	2	8031045[uid]
gyrA_A90V	OFX	2	0	2	8031045[uid]
gyrA_D94A	MOX	1	0	1	8031045[uid]
gyrA_D94A	OFX	1	0	1	8031045[uid]
gyrA_D94G	CIP	18	1	19	8031045[uid]
gyrA_D94G	MOX	9	4	13	8031045[uid]
gyrA_D94G	OFX	9	3	12	8031045[uid]
gyrA_D94N	MOX	1	0	1	8031045[uid]
gyrA_D94N	OFX	1	0	1	8031045[uid]
gyrA_S91P	CIP	2	2	4	8031045[uid]
gyrA_S91P	MOX	3	0	3	8031045[uid]
gyrA_S91P	OFX	3	0	3	8031045[uid]
inhA_I194T	INH	2	0	2	16495272[uid]
inhA_I21T	INH	2	0	2	14638486[uid]
inhA_S94A	INH	4	1	5	10815738[uid]
katG_1450_delC	INH	1	0	1	
katG_1910_delA	INH	1	0	1	
katG_471_delG	INH	2	0	2	
katG_L159P	INH	1	0	1	
katG_S315N	INH	2	1	3	9210694[uid]
katG_S315T	INH	195	1	196	8537659[uid]
katG_T180K	INH	1	0	1	16870753[uid]
katG_V633A	INH	1	1	2	
katG_W191R	INH	1	0	1	15793126[uid]
katG_W300C	INH	1	0	1	22646308[uid] **
katG_W328L	INH	1	0	1	9210694[uid]
katG_W90R	INH	1	0	1	
pncA_177_delC	PZA	1	0	1	
pncA_292_insAT	PZA	1	0	1	

pncA_409_delT	PZA	2	0	2	
pncA_491_insACC	PZA	1	0	1	
pncA_494_delC	PZA	1	0	1	
pncA_528_insGGCCGTCTGGC	PZA	1	0	1	
pncA_570_insCT	PZA	2	0	2	
pncA_A-11G	PZA	3	0	3	9056006[uid]
pncA_C138R	PZA	1	0	1	25336456[uid]
pncA_C14R	PZA	1	0	1	9055989[uid]
pncA_D12A	PZA	1	0	1	9055989[uid]
pncA_D136N	PZA	1	2	3	11641519[uid]
pncA_D49N	PZA	1	0	1	
pncA_D8G	PZA	1	0	1	11641519[uid]
pncA_D8N	PZA	1	0	1	25336456[uid]
pncA_G132D	PZA	1	0	1	9692180[uid]
pncA_G162D	PZA	1	0	1	17360809[uid]
pncA_G78C	PZA	1	0	1	
pncA_G97D	PZA	1	0	1	11083630[uid]
pncA_H57D	PZA	11	0	11	9056006[uid]
pncA_H57R	PZA	2	0	2	
pncA_K96T	PZA	1	0	1	9055989[uid]
pncA_L172P	PZA	1	0	1	9692180[uid]
pncA_L27P	PZA	1	0	1	17596354[uid]
pncA_L4S	PZA	4	0	4	11083630[uid]
pncA_Q10*	PZA	1	0	1	10390239[uid]
pncA_Q141*	PZA	1	0	1	11641519[uid]
pncA_S104R	PZA	2	0	2	9692180[uid]
pncA_T-12C	PZA	3	0	3	
pncA_V125G	PZA	4	0	4	18573039[uid]
pncA_V139L	PZA	1	0	1	11083630[uid]
pncA_V180F	PZA	1	0	1	11641519[uid]
pncA_V7L	PZA	3	0	3	
pncA_W68C	PZA	1	0	1	25336456[uid]
rpoB_1396_insATTC	RIF	2	0	2	
rpoB_1427_delTGGCCCC	RIF	1	0	1	
rpoB_D435F	RIF	1	0	1	16229229[uid]
rpoB_D435V	RIF	4	0	4	15184414[uid]
rpoB_H445D	RIF	6	0	6	14729930[uid]
rpoB_H445N	RIF	1	4	5	14729930[uid]
rpoB_H445R	RIF	3	0	3	9003625[uid]
rpoB_H445Y	RIF	12	0	12	8027320[uid]
rpoB_I491F	RIF	4	19	23	10565894[uid]
rpoB_L452P	RIF	4	4	8	10921994[uid]
rpoB_Q432K	RIF	1	0	1	8027320[uid]
rpoB_S431G	RIF	1	0	1	17360809[uid]
rpoB_S450F	RIF	4	0	4	15728936[uid]
rpoB_S450L	RIF	59	1	60	7946393[uid]

rpoB_S450W	RIF	3	1	4	7759399[uid]
rpoB_V170F	RIF	2	1	3	
rpoB_V262A	RIF	1	0	1	
rpoB_V359A	RIF	1	0	1	
rpsA_A440T	PZA	11	0	11	
rpsL_K43R	SM	24	0	24	7968530[uid]
rpsL_K88R	SM	11	0	11	7934937[uid]
rrs_A1401G	AK	5	0	5	8971706[uid]
rrs_A1401G	CAP	5	0	5	
rrs_A1401G	KAN	5	0	5	
rrs_A514C	SM	2	0	2	22943573[uid]
rrs_C513T	SM	2	0	2	
rrs_C517T	SM	2	0	2	15567277[uid]
tlyA_C-83T	CAP	1	0	1	

** publication based on subset of sequences included in this study.

S8.1

Characterisation of mutations previously identified in the literature as resistance-determinants. The 'literature' was defined as any mutation listed in the Dream TB database project (https://tbdreamdb.ki.se/Info/),[4] any mutation contained on one of the three line-probe assays (which also cover those identified by the Cepheid MTB/RIF Xpert). An additional manual search was also performed for any of the 120 resistance-determinants that did not match any of these sources. Analysing the literature like this of course introduces bias, as we look harder for evidence of the resistance-determining mutations in the literature than we do for the other mutations. However, that lineage-defining mutations have been characterised as resistance-determinants in the published literature at all is evidence of noise, only more of which would be found by a further manual trawl for mutations. Each mutation is counted for each drug it is associated with (i.e. some mutations are counted more than once).

Mutation	Drug	Characterisation
pncA_S104R	PZA	Resistance-determinant
gidB_R137W	SM	Resistance-determinant
katG_W328L	INH	Resistance-determinant
pncA_K96T	PZA	Resistance-determinant
rpoB_H445R	RIF	Resistance-determinant
rpsL_K43R	SM	Resistance-determinant
rpoB_S450F	RIF	Resistance-determinant
rpoB_D435V	RIF	Resistance-determinant
gyrA_D94A	OFX	Resistance-determinant
fabG1_T-8C	INH	Resistance-determinant
embB_M306I	EMB	Resistance-determinant
gidB_A200E	SM	Resistance-determinant
pncA_C138R	PZA	Resistance-determinant
rpoB_Q432K	RIF	Resistance-determinant

pncA_V180F	PZA	Resistance-determinant
embB_G406D	EMB	Resistance-determinant
rpoB_D435F	RIF	Resistance-determinant
pncA_W68C	PZA	Resistance-determinant
embB_D354A	EMB	Resistance-determinant
gyrA_S91P	MOX	Resistance-determinant
inhA_S94A	INH	Resistance-determinant
embB_Q497R	EMB	Resistance-determinant
pncA_V139L	PZA	Resistance-determinant
gyrA_D94G	CIP	Resistance-determinant
fabG1_C-15T	INH	Resistance-determinant
pncA_L4S	PZA	Resistance-determinant
rpoB_H445D	RIF	Resistance-determinant
katG_S315N	INH	Resistance-determinant
rpoB_S450W	RIF	Resistance-determinant
pncA_G162D	PZA	Resistance-determinant
pncA_D8N	PZA	Resistance-determinant
gyrA_D94N	MOX	Resistance-determinant
pncA_C14R	PZA	Resistance-determinant
rrs_A1401G	KAN	Resistance-determinant
pncA_A-11G	PZA	Resistance-determinant
rpoB_S431G	RIF	Resistance-determinant
rpsL_K88R	SM	Resistance-determinant
pncA_Q141*	PZA	Resistance-determinant
pncA_L172P	PZA	Resistance-determinant
gyrA_S91P	OFX	Resistance-determinant
pncA_G97D	PZA	Resistance-determinant
rpoB_L452P	RIF	Resistance-determinant
pncA_G132D	PZA	Resistance-determinant
fabG1_G-17T	INH	Resistance-determinant
rrs_A514C	SM	Resistance-determinant
rrs_C517T	SM	Resistance-determinant
katG_T180K	INH	Resistance-determinant
rrs_A1401G	CAP	Resistance-determinant
gidB_A134E	SM	Resistance-determinant
gyrA_D94N	OFX	Resistance-determinant
katG_S315T	INH	Resistance-determinant
pncA_H57D	PZA	Resistance-determinant
rpoB_I491F	RIF	Resistance-determinant
embB_M306V	EMB	Resistance-determinant
gyrA_D94A	MOX	Resistance-determinant
gidB_A80P	SM	Resistance-determinant
rrs_A1401G	AK	Resistance-determinant
rpoB_H445N	RIF	Resistance-determinant
pncA_D12A	PZA	Resistance-determinant
embB_G406S	EMB	Resistance-determinant

inhA_I194T	INH	Resistance-determinant
embB_G406A	EMB	Resistance-determinant
gyrA_A74S	CIP	Resistance-determinant
rpoB_S450L	RIF	Resistance-determinant
pncA_D8G	PZA	Resistance-determinant
gyrA_A90V	MOX	Resistance-determinant
rpoB_H445Y	RIF	Resistance-determinant
inhA_I21T	INH	Resistance-determinant
gyrA_S91P	CIP	Resistance-determinant
pncA_D136N	PZA	Resistance-determinant
pncA_L27P	PZA	Resistance-determinant
pncA_V125G	PZA	Resistance-determinant
eis_G-10A	KAN	Resistance-determinant
gyrA_D94G	OFX	Resistance-determinant
katG_W191R	INH	Resistance-determinant
pncA_Q10*	PZA	Resistance-determinant
gyrA_D94G	MOX	Resistance-determinant
embB_Q497K	EMB	Resistance-determinant
gyrA_A90V	OFX	Resistance-determinant
embR_C110Y	EMB	Lineage-defining
katG_R463L	INH	Lineage-defining
rmlD_S257P	EMB	Lineage-defining
embC_V981L	EMB	Lineage-defining
manB_D152N	EMB	Lineage-defining
gidB_L16R	SM	Lineage-defining
gyrA_S95T	OFX	Lineage-defining
embC_N394D	EMB	Lineage-defining
embC_R738Q	EMB	Lineage-defining
ndh_V18A	INH	Lineage-defining
embA_P913S	EMB	Lineage-defining
inhA_V78A	INH	Lineage-defining
gyrA_S95T	CIP	Lineage-defining
gyrA_T80A	MOX	Lineage-defining
gyrA_S95T	MOX	Lineage-defining
gyrA_T80A	OFX	Lineage-defining
gidB_E92D	SM	Lineage-defining
embB_E378A	EMB	Lineage-defining
embC_T270I	EMB	Lineage-defining
gyrA_T80A	CIP	Lineage-defining
iniC_P248A	EMB	Benign
ahpC_C-52T	INH	Benign
gyrA_D94H	MOX	Benign
rpoB_L430P	RIF	Benign
pncA_Y64D	PZA	Benign

pncA_V21G	PZA	Benign
iniA_S501W	EMB	Benign
pncA_L35R	PZA	Benign
ndh_R268H	INH	Benign
gyrA_D94H	CIP	Benign
pncA_A146T	PZA	Benign
embA_A201T	EMB	Benign
pncA_T47A	PZA	Benign
embB_M306L	EMB	Benign
gyrA_A90G	CIP	Benign
rpoB_D435Y	RIF	Benign
gyrA_A90G	OFX	Benign
embC_A307T	EMB	Benign
embB_F285L	EMB	Benign
pncA_V139A	PZA	Benign
rmlD_G-71T	EMB	Benign
katG_T394A	INH	Benign
embA_C-16T	EMB	Benign
gyrA_A90G	MOX	Benign
embB_L370R	EMB	Benign
embB_P430L	EMB	Benign
pncA_H71Y	PZA	Benign
pncA_T87M	PZA	Benign
gyrA_D94H	OFX	Benign
katG_M257I	INH	Benign
gidB_S70R	SM	Benign
embB_M306T	EMB	Uncharacterised
fabG1_T-8A	INH	Uncharacterised
rpsL_K88T	SM	Uncharacterised
gyrA_A90V	CIP	Uncharacterised
embA_C-12T	EMB	Uncharacterised
rpoB_I480V	RIF	Uncharacterised
ahpC_E76K	INH	Uncharacterised
fabG1_T-8G	INH	Uncharacterised
embA_C-11A	EMB	Uncharacterised
ahpC_G-74A	INH	Uncharacterised
inhA_I21V	INH	Uncharacterised
katG_D695A	INH	Uncharacterised
ahpC_G-48A	INH	Uncharacterised
ahpC_C-54T	INH	Uncharacterised
katG_Y155S	INH	Uncharacterised
ahpC_D73H	INH	Uncharacterised

S9

Genotypic predictions for all drugs for the training-set itself, and for the validation-set. Genotypic predictions based on: R (resistance-determinant); Rx (resistance-determinant as a mixed base-call); S0 (zero mutations present); Ss (only benign mutations present); U (uncharacterised mutations present). Weighted mean sensitivity and specificity presented as both an overall figure and as that based on characterised mutations only (R, Rx, S0, Ss columns). See below.

Training-set	Phenotypically Resistant						Phenotypically Sensitive						All		Excluding Unclassfied		
	Genotype						Genotype										
	R	R_x	S_0	S_s	U	Total	R	R_x	S_0	S_s	U	Total	Sensitivity	Specificity	Sensitivity	Specificity	% Unclassifed
**Isoniazid**	290	5	18	0	2	315	12	2	1601	156	1	1772	93.7	99.2	94.2	99.2	0.1
**Rifampicin**	108	1	1	0	0	110	27	3	1833	62	16	1941	99.1	98.5	99.1	98.4	0.8
**Ethambutol**	51	3	1	1	3	59	52	2	1350	567	46	2017	91.5	97.3	96.4	97.3	2.4
**Pyrazinamide**	56	4	7	0	0	67	2	0	1867	100	0	1969	89.6	99.9	89.6	99.9	0.0
**Streptomycin**	65	1	4	0	3	73	11	0	359	61	2	433	90.4	97.5	94.3	97.4	1.0
**Ofloxacin**	15	1	1	0	0	17	3	0	82	34	0	119	94.1	97.5	94.1	97.5	0.0
**Amikacin**	5	0	0	0	0	5	0	0	57	39	16	112	100.0	100.0	100.0	100.0	13.7
Total	590	15	32	1	8	646	107	7	7149	1019	81	8363	93.7	98.6	94.8	98.6	1.0
**Other fluroquinolones:**																	
Ci: Ciprofloxacin	21	1	0	0	1	23	3	0	232	53	1	289					
M: Moxifloxacin	15	1	0	0	0	16	4	0	83	34	0	121					
**Other aminoglycosides:**																	
C: Capreomycin	6	0	1	0	0	7	0	0	54	36	14	104					
K: Kanamycin	9	0	0	0	0	9	0	0	48	38	15	101					

Validation-set	R	R_x	S_0	S_s	U	Total	R	R_x	S_0	S_s	U	Total	Sensitivity	Specificity	Sensitivity	Specificity	% Unclassifed
**Isoniazid**	305	5	18	1	35	364	19	0	1,065	52	52	1188	85.2	98.4	94.2	98.3	5.6
**Rifampicin**	263	12	8	1	16	300	9	1	1,200	4	38	1252	91.7	99.2	96.8	99.2	3.5
**Ethambutol**	152	6	7	1	26	192	62	5	1003	79	210	1359	82.3	95.1	95.2	94.2	15.2
**Pyrazinamide**	31	12	27	5	104	179	2	0	1,218	67	83	1370	24.0	99.9	57.3	99.8	12.1
**Streptomycin**	278	6	6	9	49	348	10	1	970	34	189	1204	81.6	99.1	95.0	98.9	15.3
**Ofloxacin**	2	3	4	2	0	11	0	0	489	134	38	661	45.5	100.0	45.5	100.0	5.7
**Amikacin**	36	16	5	0	2	59	1	2	427	38	140	608	88.1	99.5	91.2	99.4	21.3
Total	1067	60	75	19	232	1453	103	9	6372	408	750	7642	77.6	98.5	92.3	98.4	10.8
**Other fluroquinolones:**																	
Ci: Ciprofloxacin	0	0	0	0	0	0	0	0	1	0	0	1					
M: Moxifloxacin	3	0	2	3	0	8	0	0	287	133	25	445					
**Other aminoglycosides:**																	
C: Capreomycin	31	13	6	1	4	55	6	4	428	37	140	615					
K: Kanamycin	3	0	4	1	1	9	0	0	263	50	129	442					

S10

The performance of mutations characterised in the training-set as resistance-determinants when predicting phenotypes in the validation-set.

		Phenotypically resistant		Phenotypically sensitive		
Drug	Variant	Resistance-determinant	Resistance-determinant as a mixed-call	Resistance-determinant	Resistance-determinant as a mixed-call	Link to previous report in the literature
INH	ahpC_C-57T	1	0	0	0	N/A
INH	fabG1_C-15T	32	1	9	0	10428945[uid]
INH	fabG1_G-17T	2	0	0	0	14638486[uid]
INH	fabG1_T-8C	5	1	0	0	15793126[uid]
INH	inhA_I194T	3	1	1	0	16495272[uid]
INH	inhA_I21T	1	0	0	0	14638486[uid]
INH	inhA_S94A	0	0	2	0	10815738[uid]
INH	katG_S315N	6	0	0	0	9210694[uid]
INH	katG_S315T	271	5	8	0	8537659[uid]
INH	katG_W191R	1	0	0	0	15793126[uid]
RIF	rpoB_1396_insATTC	0	1	0	0	N/A
RIF	rpoB_D435F	1	0	0	0	16229229[uid]
RIF	rpoB_D435V	9	2	0	0	15184414[uid]
RIF	rpoB_H445D	8	1	0	0	14729930[uid]
RIF	rpoB_H445N	0	0	0	1	14729930[uid]
RIF	rpoB_H445R	2	1	0	0	9003625[uid]
RIF	rpoB_H445Y	10	5	1	0	8027320[uid]
RIF	rpoB_L452P	4	0	3	0	10921994[uid]
RIF	rpoB_Q432K	0	0	1	0	8027320[uid]
RIF	rpoB_S450L	224	7	3	0	15728936[uid]
RIF	rpoB_S450W	3	1	1	0	7759399[uid]
RIF	rpoB_V170F	1	1	0	0	N/A
EMB	embB_D354A	2	0	1	0	N/A
EMB	embB_G406A	3	0	2	0	10639358[uid]
EMB	embB_G406D	3	0	2	1	10639358[uid]
EMB	embB_G406S	1	0	0	0	10639358[uid]
EMB	embB_H1002R	2	0	1	0	22646308[uid]**
EMB	embB_M306I	38	1	27	2	9257740[uid]
EMB	embB_M306V	94	5	22	4	9257740[uid]
EMB	embB_Q497K	0	0	2	0	10639358[uid]
EMB	embB_Q497R	9	0	4	0	10639358[uid]
PZA	pncA_292_insAT	1	0	0	0	N/A
PZA	pncA_494_delC	1	0	0	0	N/A
PZA	pncA_A-11G	15	5	1	0	9056006[uid]
PZA	pncA_C138R	0	2	0	0	25336456[uid]

PZA	pncA_C14R	2	1	0	0	9055989[uid]
PZA	pncA_D136N	0	1	0	0	11641519[uid]
PZA	pncA_G97D	2	0	0	0	11083630[uid]
PZA	pncA_H57D	7	0	0	0	9056006[uid]
PZA	pncA_Q141*	1	0	0	0	11641519[uid]
PZA	pncA_T-12C	0	1	0	0	N/A
PZA	pncA_V125G	2	2	0	0	18573039[uid]
PZA	pncA_V139L	0	1	0	0	11083630[uid]
PZA	rpsA_A440T	7	0	1	0	N/A
SM	gidB_202_delG	4	0	2	0	N/A
SM	gidB_A134E	2	0	0	0	17238915[uid]
SM	gidB_V65G	1	1	0	0	N/A
SM	rpsL_K43R	229	3	7	1	7968530[uid]
SM	rpsL_K88R	16	1	0	0	7934937[uid]
SM	rrs_A514C	19	2	0	0	22943573[uid]
SM	rrs_C517T	6	1	2	0	15567277[uid]
CIP	.	.	.	.	.	
MOX	gyrA_D94A	1	0	0	0	8031045[uid]
MOX	gyrA_D94G	2	0	0	0	8031045[uid]
OFX	gyrA_A90V	0	2	0	0	8031045[uid]
OFX	gyrA_D94G	2	3	0	0	8031045[uid]
AK	rrs_A1401G	36	16	1	2	8971706[uid]
CAP	rrs_A1401G	31	13	6	4	N/A
KAN	rrs_A1401G	3	0	0	0	N/A

S11

Susceptible phenotypes predicted resistant in the validation-set

Isolate	Drug	Resistance-determinant
ERR551030	EMB	embB_D354A
ERR551894,ERR551895	EMB	embB_G406A
ERR551634,ERR551635	EMB	embB_G406A
ERR551952,ERR551951	EMB	embB_G406D
ERR552080,ERR552079	EMB	embB_G406D
ERR551409,ERR551410	EMB	embB_G406D
ERR551910,ERR551909	EMB	embB_H1002R
SAMN03649008	EMB	embB_M306I
ERR552413,ERR552412	EMB	embB_M306I
SAMN03648591	EMB	embB_M306I
ERR552940,ERR552939	EMB	embB_M306I
ERR552611,ERR552610	EMB	embB_M306I
ERR551828,ERR551829	EMB	embB_M306I
SAMN03648978	EMB	embB_M306I
ERR552298,ERR552297	EMB	embB_M306I
ERR552106,ERR552105	EMB	embB_M306I
ERR551266	EMB	embB_M306I
SAMN03649022	EMB	embB_M306I
ERR550897,ERR550896	EMB	embB_M306I
ERR552068,ERR552067	EMB	embB_M306I
ERR551771,ERR551770	EMB	embB_M306I
ERR552398,ERR552399	EMB	embB_M306I
ERR551660,ERR551659	EMB	embB_M306I
ERR552603,ERR552604	EMB	embB_M306I
SAMN03648845	EMB	embB_M306I
ERR552931,ERR552932	EMB	embB_M306I
ERR551816,ERR551815	EMB	embB_M306I
ERR552433,ERR552434	EMB	embB_M306I
ERR552677	EMB	embB_M306I
ERR551645,ERR551644	EMB	embB_M306I
SAMN03648802	EMB	embB_M306I
ERR552373,ERR552374	EMB	embB_M306I
ERR552363,ERR552362,ERR552364	EMB	embB_M306I
ERR550748,ERR550749	EMB	embB_M306I
ERR551108,ERR551109	EMB	embB_M306I
ERR550928,ERR550929	EMB	embB_M306I

ERR551377,ERR551379,ERR551378	EMB	embB_M306V
ERR552226,ERR552225	EMB	embB_M306V
SAMN03649010	EMB	embB_M306V
ERR550770,ERR550771	EMB	embB_M306V
ERR553196	EMB	embB_M306V
ERR552559,ERR552561,ERR552560	EMB	embB_M306V
ERR552080,ERR552079	EMB	embB_M306V
ERR551745,ERR551744,ERR551743	EMB	embB_M306V
ERR552950,ERR552951	EMB	embB_M306V
ERR551862,ERR551861	EMB	embB_M306V
ERR551667,ERR551668	EMB	embB_M306V
ERR550764	EMB	embB_M306V
ERR552200,ERR552199	EMB	embB_M306V
ERR552872,ERR552873,ERR552874	EMB	embB_M306V
ERR551142,ERR551141	EMB	embB_M306V
ERR553246,ERR553247	EMB	embB_M306V
ERR551643,ERR551642	EMB	embB_M306V
ERR551798,ERR551797	EMB	embB_M306V
ERR551985,ERR551984	EMB	embB_M306V
SAMN03648988	EMB	embB_M306V
ERR553098,ERR553097	EMB	embB_M306V
ERR552033,ERR552032	EMB	embB_M306V
ERR551437,ERR551436	EMB	embB_M306V
ERR553192,ERR553191	EMB	embB_M306V
ERR551464,ERR551465	EMB	embB_M306V
ERR552164,ERR552165	EMB	embB_M306V
SAMN03648554	EMB	embB_Q497K
ERR552091	EMB	embB_Q497K
ERR552326	EMB	embB_Q497R
ERR551251,ERR551250	EMB	embB_Q497R
ERR552443,ERR552442	EMB	embB_Q497R
ERR552580	EMB	embB_Q497R
SAMN03648823	INH	fabG1_C-15T
ERR551893	INH	fabG1_C-15T
ERR550956	INH	fabG1_C-15T
ERR550825	INH	fabG1_C-15T
SAMN03648742	INH	fabG1_C-15T
ERR551538	INH	fabG1_C-15T
ERR552371	INH	fabG1_C-15T
SAMN03648901	INH	fabG1_C-15T
SAMN03648707	INH	fabG1_C-15T

ERR550943	SM	gidB_202_delG
ERR552264	SM	gidB_202_delG
ERR551538	INH	inhA_I194T
ERR551632	INH	inhA_S94A
SAMN03648620	INH	inhA_S94A
ERR552831	INH	katG_S315T
SAMN03648727	INH	katG_S315T
ERR551327	INH	katG_S315T
ERR553196	INH	katG_S315T
ERR552694	INH	katG_S315T
SAMN03648562	INH	katG_S315T
ERR553262	INH	katG_S315T
ERR551232	INH	katG_S315T
ERR552301,ERR552302	PZA	pncA_A-11G
SAMN03648779	RIF	rpoB_H445N
ERR552872,ERR552873,ERR552874	RIF	rpoB_H445Y
SAMN03648786	RIF	rpoB_L452P
SAMN03648884	RIF	rpoB_L452P
ERR552539	RIF	rpoB_L452P
SAMN03648559	RIF	rpoB_Q432K
SAMN03649015	RIF	rpoB_S450L
ERR553196	RIF	rpoB_S450L
SAMN03648974	RIF	rpoB_S450L
ERR550793	RIF	rpoB_S450W
ERR552130	SM	rpsL_K43R
ERR553196	SM	rpsL_K43R
ERR552683	SM	rpsL_K43R
ERR552892	SM	rpsL_K43R
ERR551859	SM	rpsL_K43R
ERR551119	SM	rpsL_K43R
SAMN03648559	SM	rpsL_K43R
ERR551232	SM	rpsL_K43R
ERR550773,ERR550772	AK	rrs_A1401G
ERR552148,ERR552147	AK	rrs_A1401G

ERR552872,ERR552873,ERR552874	AK	rrs_A1401G
SAMN03649053	SM	rrs_C517T
ERR553196	SM	rrs_C517T

S12

Comparison on derived training-set mutations to the *in silico* performance of line-probe assay (LPA) based mutations. Results are shown for the LPA mutations, then for the derived mutations (for the same drugs), and then for the combined mutations from the LPAs and the training-set. Genotypic predictions based on: R (resistance-determinant); Rx (resistance-determinant as a mixed base-call); S0 (zero mutations present); Ss (only benign mutations present); U (uncharacterised mutations present). Weighted mean sensitivity and specificity presented for a subset of drugs to avoid double counting. Ofloxacin and amikacin were included as representatives of their antibiotic classes as these had more resistant phenotypes than their related drugs. Pyrazinamide excluded for line-probe comparison as no line-probe assay exists for pyrazinamide (see below).

Results for line-probe assay alleles

	Phenotypically Resistant Genotype						Phenotypically Sensitive Genotype						All		Excluding Unclassfied	
	R	R_x	S_0	S_s	U	Total	R	R_x	S_0	S_s	U	Total	Sensitivity	Specificity		
Isoniazid	304	5	0	55	0	364	17	0	0	1171	0	1188	84.9	98.6		
Rifampicin	272	14	0	14	0	300	33	0	0	1219	0	1252	95.3	97.4		
Ethambutol	132	6	0	54	0	192	53	6	0	1300	0	1359	71.9	95.7		
Streptomycin	246	2	0	100	0	348	7	1	0	1196	0	1204	71.3	99.3		
Ofloxacin	3	3	0	5	0	11	5	0	0	656	0	661	54.5	99.2		
Amikacin	37	16	0	6	0	59	2	2	0	604	0	608	89.8	99.3		
Total	994	46	0	234	0	1274	117	9	0	6146	0	6272	81.6	98.0		

Results for training-set

	R	R_x	S_0	S_s	U	Total	R	R_x	S_0	S_s	U	Total	Sensitivity	Specificity	Sensitivity	Specificity
Isoniazid	305	5	18	1	35	364	19	0	1,065	52	52	1188	85.2	98.4	94.2	98.3
Rifampicin	263	12	8	1	16	300	9	1	1,200	4	38	1252	91.7	99.2	96.8	99.2
Ethambutol	152	6	7	1	26	192	62	5	1003	79	210	1359	82.3	95.1	95.2	94.2
Streptomycin	278	6	6	9	49	348	10	1	970	34	189	1204	81.6	99.1	95.0	98.9
Ofloxacin	2	3	4	2	0	11	0	0	489	134	38	661	45.5	100.0	45.5	100.0
Amikacin	36	16	5	0	2	59	1	2	427	38	140	608	88.1	99.5	91.2	99.4
Total	1036	48	48	14	128	1274	101	9	5154	341	667	6272	85.1	98.2	94.6	98.0

Results for combined alleles from training-set and line-probe assays

	R	R_x	S_0	S_s	U	Total	R	R_x	S_0	S_s	U	Total	Sensitivity	Specificity	Sensitivity	Specificity
Isoniazid	306	5	18	1	34	364	19	0	1,065	52	52	1188	85.4	98.4	94.2	98.3
Rifampicin	273	13	8	0	6	300	12	3	1,200	2	35	1252	95.3	98.8	97.3	98.8
Ethambutol	152	6	7	1	26	192	65	6	1003	77	208	1359	82.3	94.8	95.2	93.8
Pyrazinamide	31	12	27	5	104	179	2	0	1,218	67	83	1370	24.0	99.9	57.3	99.8
Streptomycin	278	6	6	9	49	348	10	1	970	34	189	1204	81.6	99.1	95.0	98.9
Ofloxacin	3	3	4	1	0	11	0	0	489	134	38	661	54.5	100.0	54.5	100.0
Amikacin	37	16	5	0	1	59	2	2	427	38	139	608	89.8	99.3	91.4	99.1
Total	1080	61	75	17	220	1453	110	12	6372	404	744	7642	78.5	98.4	92.5	98.2
												Excluding pyrazinamide:	86.2	98.1	94.8	97.9

S13

Characterisation of mutations by the original training-set and by all 3651 samples.

Previously seen in the literature	Characterisation in the training-set	Characterisation in the final, combined set of 3651	Which isolates was the final characterisation based on?	Number of mutations
No	Mutation not seen	Resistance-determinant	Validation-set	71
Yes	Mutation not seen	Resistance-determinant	Validation-set	26
No	Mutation not seen	Benign	Validation-set	872
Yes	Mutation not seen	Benign	Validation-set	14
No	Mutation not seen	Uncharacterised	Validation-set	528
Yes	Mutation not seen	Uncharacterised	Validation-set	8
No	Resistance-determinant	Resistance-determinant	Training-set	30
Yes	Resistance-determinant	Resistance-determinant	Training-set	30
No	Resistance-determinant	Resistance-determinant	Combined sets	8
Yes	Resistance-determinant	Resistance-determinant	Combined sets	48
No	Resistance-determinant	Benign	Combined sets	2
No	Resistance-determinant	Uncharacterised	Training-set	1
Yes	Resistance-determinant	Uncharacterised	Training-set	1
No	Benign	Resistance-determinant	Combined sets	9
Yes	Benign	Resistance-determinant	Combined sets	7
No	Benign	Benign	Training-set	607
Yes	Benign	Benign	Training-set	13
No	Benign	Benign	Combined sets	113
Yes	Benign	Benign	Combined sets	11
No	Benign	Uncharacterised	Combined sets	12
No	Uncharacterised	Resistance-determinant	Combined sets	2
Yes	Uncharacterised	Resistance-determinant	Combined sets	1
No	Uncharacterised	Benign	Combined sets	2
Yes	Uncharacterised	Benign	Combined sets	1
No	Uncharacterised	Uncharacterised	Training-set	57
Yes	Uncharacterised	Uncharacterised	Training-set	9
No	Uncharacterised	Uncharacterised	Combined sets	24
Yes	Uncharacterised	Uncharacterised	Combined sets	5

S14

Resistance-determinants algorithmically derived from the 3651 isolates

Mutation	Drug	Resistant	Susceptible
ahpC_C-57T	INH	2	0
ahpC_C-72T	INH	1	1
eis_G-10A	KAN	4	0
embA_C-12T	EMB	12	7
embA_C-16G	EMB	5	1
embA_C-16T	EMB	6	4
embB_D328Y	EMB	2	1
embB_D354A	EMB	3	2
embB_G406A	EMB	4	5
embB_G406D	EMB	5	5
embB_G406S	EMB	4	5
embB_M306I	EMB	59	63
embB_M306V	EMB	117	29
embB_N1033K	EMB	1	0
embB_Q497K	EMB	1	4
embB_Q497R	EMB	17	8
fabG1_C-15T	INH	106	15
fabG1_G-17T	INH	7	1
fabG1_T-8C	INH	9	3
gidB_141_delC	SM	1	0
gidB_202_delG	SM	8	9
gidB_202_insGC	SM	1	1
gidB_215_delC	SM	6	3
gidB_399_delAAACTCGGTG	CAP	1	0
gidB_399_delAAACTCGGTG	SM	1	0
gidB_451_delG	SM	4	4
gidB_451_insGC	SM	1	2
gidB_A134E	SM	3	0
gidB_A138T	SM	1	0
gidB_A138V	SM	2	1
gidB_A19P	SM	1	2
gidB_A200E	SM	2	0
gidB_A205E	SM	1	0
gidB_A80P	SM	1	0
gidB_C52F	SM	1	0
gidB_D85A	SM	3	0
gidB_E173*	SM	1	0
gidB_G117V	SM	2	0
gidB_G30D	SM	4	0
gidB_G34V	SM	2	0
gidB_G69D	SM	3	0
gidB_G73A	SM	1	0

gidB_H48N	SM	2	0
gidB_H48Q	SM	1	0
gidB_I11N	SM	1	0
gidB_I162S	SM	1	0
gidB_L26F	SM	1	0
gidB_L79S	SM	5	2
gidB_L79W	SM	1	0
gidB_L91P	SM	1	0
gidB_P75L	SM	1	1
gidB_P75R	SM	5	0
gidB_P93L	SM	1	0
gidB_Q125*	SM	1	0
gidB_R118L	SM	1	0
gidB_R118S	SM	3	1
gidB_R137P	SM	2	1
gidB_R137W	SM	1	1
gidB_R47W	SM	1	0
gidB_R64W	SM	1	0
gidB_R83P	SM	1	0
gidB_S136*	SM	1	1
gidB_S149R	SM	1	2
gidB_S70N	SM	1	0
gidB_V203L	SM	1	0
gidB_V41I	SM	1	1
gidB_V65G	SM	3	0
gidB_V88A	SM	2	0
gidB_Y195H	CAP	2	28
gyrA_A74S	CIP	2	0
gyrA_A90V	MOX	2	0
gyrA_A90V	OFX	4	0
gyrA_D94A	MOX	2	0
gyrA_D94A	OFX	1	0
gyrA_D94G	CIP	18	1
gyrA_D94G	MOX	11	4
gyrA_D94G	OFX	14	3
gyrA_D94H	MOX	1	1
gyrA_D94H	OFX	1	1
gyrA_D94N	MOX	1	0
gyrA_D94N	OFX	1	0
gyrA_S91P	CIP	2	2
gyrA_S91P	MOX	3	0
gyrA_S91P	OFX	3	0
inhA_I194T	INH	6	1
inhA_I21T	INH	3	0
inhA_S94A	INH	4	3
katG_1157_delT	INH	1	0

katG_121_insTA	INH	1	0
katG_1388_delA	INH	2	0
katG_1450_delC	INH	1	0
katG_1465_delC	INH	1	0
katG_1910_delA	INH	1	0
katG_471_delG	INH	2	0
katG_A109V	INH	1	0
katG_A614E	INH	1	0
katG_D142G	INH	1	0
katG_G125D	INH	1	0
katG_G182R	INH	1	0
katG_G297V	INH	1	0
katG_L141F	INH	3	0
katG_L159P	INH	1	0
katG_L627P	INH	1	0
katG_L704S	INH	1	0
katG_P232R	INH	1	0
katG_R104Q	INH	2	0
katG_S315I	INH	1	0
katG_S315N	INH	8	1
katG_S315T	INH	471	9
katG_S481L	INH	1	1
katG_S700P	INH	2	0
katG_T180K	INH	1	0
katG_V633A	INH	1	1
katG_W191G	INH	1	0
katG_W191R	INH	2	0
katG_W300C	INH	1	0
katG_W300S	INH	1	0
katG_W328L	INH	1	0
katG_W505*	INH	1	0
katG_W90R	INH	1	0
ndh_G225D	INH	1	0
pncA_174_delG	PZA	1	0
pncA_177_delC	PZA	1	0
pncA_285_insCT	PZA	1	0
pncA_292_insAT	PZA	2	0
pncA_409_delT	PZA	2	0
pncA_489_delC	PZA	9	0
pncA_491_insACC	PZA	1	0
pncA_494_delC	PZA	2	0
pncA_528_insGGCCGTCTGGC	PZA	1	0
pncA_556_insTG	PZA	1	0
pncA_563_insAC	PZA	1	0
pncA_570_insCT	PZA	2	0
pncA_592_delC	PZA	1	0

pncA_617_insTC	PZA	1	0
pncA_A-11G	PZA	23	1
pncA_C138R	PZA	3	0
pncA_C14R	PZA	4	0
pncA_D12A	PZA	1	0
pncA_D136N	PZA	2	2
pncA_D49N	PZA	1	0
pncA_D8G	PZA	1	0
pncA_D8N	PZA	1	0
pncA_F81V	PZA	1	1
pncA_G132D	PZA	1	0
pncA_G162D	PZA	1	0
pncA_G78C	PZA	1	0
pncA_G97C	PZA	1	0
pncA_G97D	PZA	3	0
pncA_G97R	PZA	1	0
pncA_H137R	PZA	1	2
pncA_H51Q	PZA	1	1
pncA_H57D	PZA	18	0
pncA_H57R	PZA	2	0
pncA_H71Q	PZA	1	0
pncA_H71Y	PZA	2	1
pncA_I133T	PZA	20	15
pncA_K48E	PZA	1	2
pncA_K96T	PZA	1	0
pncA_L151S	PZA	1	0
pncA_L159V	PZA	1	1
pncA_L172P	PZA	1	0
pncA_L27P	PZA	1	0
pncA_L4S	PZA	4	0
pncA_M175V	PZA	1	1
pncA_P54L	PZA	2	2
pncA_P54Q	PZA	1	1
pncA_Q10*	PZA	1	0
pncA_Q10P	PZA	38	3
pncA_Q141*	PZA	2	0
pncA_Q141P	PZA	5	1
pncA_S104G	PZA	1	1
pncA_S104R	PZA	2	0
pncA_S32I	PZA	1	0
pncA_T-12C	PZA	4	0
pncA_T114P	PZA	1	0
pncA_T135P	PZA	1	0
pncA_T47A	PZA	1	1
pncA_V125G	PZA	8	0
pncA_V139L	PZA	2	0

pncA_V180F	PZA	1	0
pncA_V180G	PZA	2	0
pncA_V21G	PZA	2	1
pncA_V7L	PZA	3	0
pncA_W68C	PZA	1	0
pncA_W68R	PZA	4	0
pncA_Y99*	PZA	1	0
rpoB_1377_delG	RIF	1	0
rpoB_1392_insGCCA	RIF	2	0
rpoB_1394_delC	RIF	1	0
rpoB_1396_insATTC	RIF	3	0
rpoB_1398_delT	RIF	1	0
rpoB_1427_delTGGCCCC	RIF	1	0
rpoB_D435F	RIF	2	0
rpoB_D435V	RIF	15	0
rpoB_G981D	RIF	1	0
rpoB_H445D	RIF	15	0
rpoB_H445L	RIF	2	0
rpoB_H445N	RIF	1	5
rpoB_H445R	RIF	6	0
rpoB_H445Y	RIF	27	1
rpoB_I491F	RIF	4	19
rpoB_L430P	RIF	3	4
rpoB_L452P	RIF	8	7
rpoB_M434I	RIF	1	0
rpoB_Q432K	RIF	1	1
rpoB_Q432L	RIF	1	0
rpoB_Q432P	RIF	2	0
rpoB_S441L	RIF	1	0
rpoB_S450F	RIF	4	0
rpoB_S450L	RIF	290	4
rpoB_S450W	RIF	7	2
rpoB_T676P	RIF	1	0
rpoB_V170F	RIF	4	1
rpoB_V359A	RIF	1	0
rpsA_E67D	PZA	1	0
rpsA_V260I	PZA	1	16
rpsL_K43R	SM	256	8
rpsL_K88R	SM	28	0
rrs_A1325C	SM	1	0
rrs_A1401G	AK	57	3
rrs_A1401G	CAP	49	10
rrs_A1401G	KAN	8	0
rrs_A514C	SM	23	0
rrs_A514T	SM	1	0
rrs_C1402T	AK	1	1

rrs_C1402T	CAP	2	1
rrs_C513T	SM	2	0
rrs_C517T	KAN	1	5
rrs_C517T	SM	9	2
rrs_C905A	SM	1	0
tlyA_C-83T	CAP	1	0

S15

Algorithm as applied to all 3651 isolates

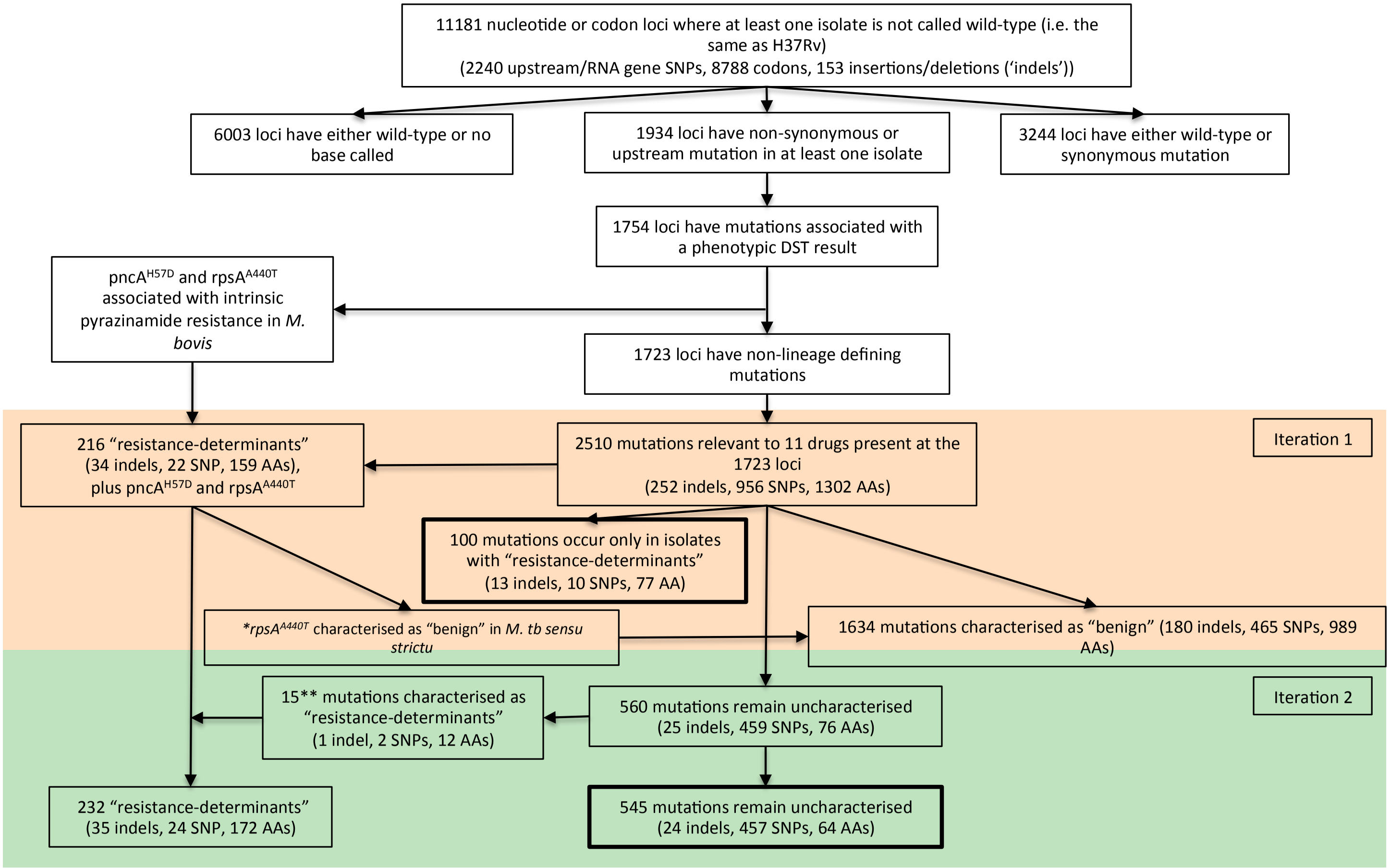

*Although in the training-set *rpsA*^{A440T} was identified as lineage-defining of *M. bovis*, which is intrinsically resistant to pyrazinamide, it is seen independently as a lone-standing (non- *M. bovis*) mutation in a pyrazinamide sensitive isolate in the validation-set, leading to its re-characterised as "benign", and leaving *pncA*^{H57D} as the remaining resistance-determinant for *M. bovis*. 645 mutations were left uncharacterised (highlighted in boxes in bold). **Mutations occurring in phenotypically-resistant isolates with the other mutations defined as 'benign' in iteration 1.

S16

Phenotypic predictions for all 3651 isolates, based on mutations derived from applying the algorithm to the same 3651 isolates.

	Phenotypically Resistant						Phenotypically Resistant						All		Excluding Unclassfied	
	Genotype						Genotype						Sensitivity	Specificity	Sensitivity	Specificity
	R	R_x	S_0	S_s	U	Total	R	R_x	S_0	S_s	U	Total				
**H: Isoniazid**	621	12	36	0	10	**679**	33	2	2,666	244	15	**2960**	**93.2**	**98.8**	**94.6**	**98.8**
**R: Rifampicin**	385	14	9	0	2	**410**	40	4	3,033	91	25	**3193**	**97.3**	**98.6**	**97.8**	**98.6**
**E: Ethambutol**	217	10	8	0	16	**251**	122	8	2,353	645	248	**3376**	**90.4**	**96.1**	**96.6**	**95.8**
**Z: Pyrazinamide**	187	24	34	0	1	**246**	47	5	3,085	196	6	**3339**	**85.8**	**98.4**	**86.1**	**98.4**
**S: Streptomycin**	397	7	10	0	7	**421**	40	1	1,329	127	140	**1637**	**96.0**	**97.5**	**97.6**	**97.3**
**O: Ofloxacin**	18	4	5	0	1	**28**	4	0	571	120	85	**780**	**78.6**	**99.5**	**81.5**	**99.4**
**A: Amikacin**	42	16	5	0	1	**64**	2	2	484	75	157	**720**	**90.6**	**99.4**	**92.1**	**99.3**
**Total**	1867	87	107	0	38	2099	288	22	13521	1498	676	16005	**93.1**	**98.1**	**94.8**	**98.0**
**Other quinilones**																
Ci: Ciprofloxacin	21	1	0	0	1	23	3	0	233	42	12	290				
M: Moxifloxacin	19	1	2	0	2	24	5	0	370	108	83	566				
**Other aminoglycosides**																
C: Capreomycin	41	13	7	0	1	62	35	4	482	72	126	719				
K: Kanamycin	13	0	4	0	1	18	5	0	311	80	147	543				

Genotypic predictions for all phenotypes for all 3651 isolates. Genotypic predictions based on: R (resistance-determinant); Rx (resistance-determinant as a mixed base-call); S0 (zero mutations present); Ss (only benign mutations present); U (uncharacterised mutations present). Weighted mean sensitivity and specificity presented as both an overall figure and as that based on characterised mutations only (R, Rx, S0, Ss columns)

S17

Homoplasy rates vs. the proportion of phenotypic-resistance for mutations in the 23-candidate genes and the rest of the genome.

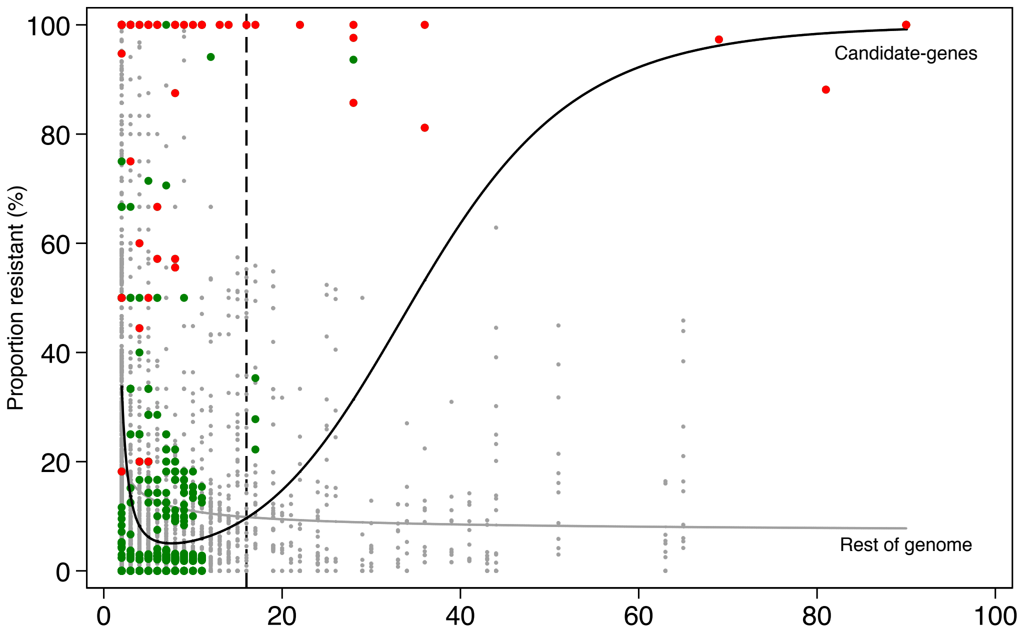

Mutations within the candidate-genes are shown as red (characterised as resistance-determinants) and green (others). Mutations in the rest of the genome are shown in grey. Curves represent the mean for mutations across candidate-genes and across the rest of the genome. The mutation katG^{S315T} emerged 180 times but has been set to 90, equal to the next most homoplastic mutation, to fit all data to the current scale. The proportion resistant has not been changed and shape of the curve of the mean for candidate-genes remains unaltered.

References

1. Walker TM, Lalor MK, Broda A, Saldana Ortega L, Morgan M, Parker L, et al. Assessment of Mycobacterium tuberculosis transmission in Oxfordshire, UK, 2007-12, with whole pathogen genome sequences: an observational study. The Lancet Respiratory Medicine. 2014 Apr;2(4):285–92.

2. Walker TM, Ip CL, Harrell RH, Evans JT, Kapatai G, Dedicoat MJ, et al. Whole-genome sequencing to delineate Mycobacterium tuberculosis outbreaks: a retrospective observational study. Lancet Infect Dis. 2013 Feb;13(2):137–46.

3. Feuerriegel S, Oberhauser B, George A, Dafae F, Richter E, Rüsch-Gerdes S, et al. Sequence analysis for detection of first-line drug resistance in Mycobacterium tuberculosis strains from a high-incidence setting. BMC Microbiol. 2012 Jan 1;12(1):90–0.

4. Sandgren A, Strong M, Muthukrishnan P, Weiner BK, Church GM, Murray MB. Tuberculosis Drug Resistance Mutation Database. Plos Med. 2009;6(2):e2.
